# Utilizing RNA origami scaffolds in *Saccharomyces cerevisiae* for dCas9-mediated transcriptional control

**DOI:** 10.1093/nar/gkac470

**Published:** 2022-06-01

**Authors:** Georgios Pothoulakis, Michael T A Nguyen, Ebbe S Andersen

**Affiliations:** Interdisciplinary Nanoscience Center, Aarhus University, 8000 Aarhus C, Denmark; Interdisciplinary Nanoscience Center, Aarhus University, 8000 Aarhus C, Denmark; Interdisciplinary Nanoscience Center, Aarhus University, 8000 Aarhus C, Denmark; Department of Molecular Biology and Genetics, Aarhus University, 8000 Aarhus C, Denmark

## Abstract

Designer RNA scaffolds constitute a promising tool for synthetic biology, as they can be genetically expressed to perform specific functions *in vivo* such as scaffolding enzymatic cascades and regulating gene expression through CRISPR-dCas9 applications. RNA origami is a recently developed RNA design approach that allows construction of large RNA nanostructures that can position aptamer motifs to spatially organize other molecules, including proteins. However, it is still not fully understood how positioning multiple aptamers on a scaffold and the orientation of a scaffold affects functional properties. Here, we investigate fusions of single-guide RNAs and RNA origami scaffolds (termed sgRNAO) capable of recruiting activating domains for control of gene expression in yeast. Using MS2 and PP7 as orthogonal protein-binding aptamers, we observe a gradual increase in transcriptional activation for up to four aptamers. We demonstrate that different aptamer positions on a scaffold and scaffold orientation affect transcriptional activation. Finally, sgRNAOs are used to regulate expression of enzymes of the violacein biosynthesis pathway to control metabolic flux. The integration of RNA origami nanostructures at promoter sites achieved here, can in the future be expanded by the addition of functional motifs such as riboswitches, ribozymes and sensor elements to allow for complex gene regulation.

## INTRODUCTION

A core goal of synthetic biology is the development of new molecular tools that enable complex gene regulation. Several approaches have been proposed, including the utilization of transcription activator-like effectors (TALEs), zinc-finger proteins and catalytically dead Cas9 (dCas9) regulation for both activation and repression of gene expression through the recruitment of activating or repressing protein domains (1–4). The advantage of dCas9-mediated regulation of target loci is the fact that specificity is solely controlled by CRISPR single-guide RNAs (sgRNAs), which are easy to design and express in cells, provide high efficiency and also have the ability to multiplex (5). The ease of sgRNA sequence design for gene regulation has also been demonstrated for bacterial and mammalian cells through the development of conditional guide RNAs (cgRNAs) which are activated by unique RNA triggers (6,7).

Further expansion of dCas9-mediated transcriptional regulation to complex metabolic pathway control is of considerable interest. Zalatan *et al.* extended the CRISPR-dCas9 system's regulatory capacity by designing sgRNA-based scaffolds (scRNA) which incorporate protein-binding RNA motifs capable of both locus targeting and additional regulatory action (8). This was achieved, through direct recruitment of corresponding activating or repressing protein domains thus allowing for the creation of scRNA programs that regulate multiple genetic targets upon induction of dCas9. Essentially, dCas9 was proposed as a master controller of complex pathway regulation in yeast and mammalian cells. This work was eventually expanded to bacterial systems where the availability of gene activators is limited (9). Further substantiating the significance of sgRNA sequence expansion with other RNA motifs, Shechner *et al.* developed strategies in mammalian cells for the insertion of long non-coding RNAs (lncRNA) several kilobases in length in sgRNAs (10).

RNA nanotechnology advancements have showcased the ability of rationally designing artificial RNA molecules capable of assembling into functional 2D scaffolds through the utilization of discrete RNA structural modules (11,12). RNA nanoparticles provide a clear advantage over DNA-based nanostructures when it comes to *in vivo* applications since they can be genetically encoded, expressed and self-assemble in cells, and incorporate several functional RNA motifs (13–16). Accessory RNA motifs, such as aptamers from the MS2 and PP7 bacteriophages which bind to their respective coat proteins, have been used to enable both gene regulation and assemble proteins *in vivo* (10,17,18). To improve our ability to rationally design RNA scaffolds, a technology for the design of co-transcriptionally assembled RNA scaffolds from a single RNA strand, termed RNA origami, was developed, assisted by computational design tools (19,20). RNA origami provides several advantages such as their high folding yield, large size and resistance to hydrolysis and degradation (20–22). Its well-defined structure improvs folding of integrated RNA functional motifs and controls their spatial positioning (23). Fusing RNA scaffolds on sgRNAs has previously been shown to be beneficial over combining tandem protein-binding aptamers on unstructured strands (8).

**Figure 1.**
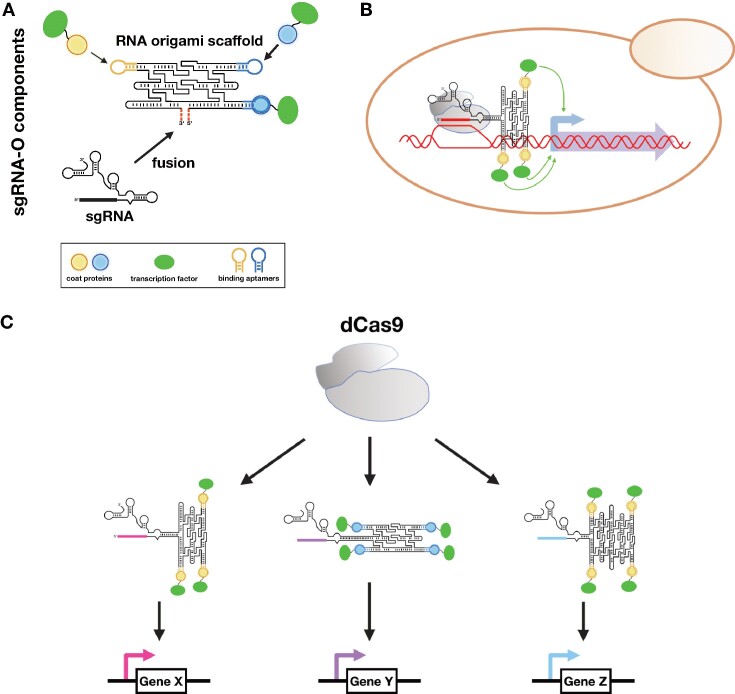
Utlizing sgRNAOs for complex transcriptional regulation in *S. cerevisiae*. (**A**) Schematics of the components used to design sgRNAOs carrying aptamers for binding of coat proteins fused to transcription factors. The initial design stage involves the selection of well-characterized functional RNA motifs (e.g., protein-binding aptamers) and their integration to an appropriate RNA origami scaffold. RNA origami scaffolds are subsequently fused to the engineered loop of an sgRNA. Final RNA sequences targeting specific 2D structure targets are generated using RNA origami computational tools. (**B**) sgRNAO for indirect gene regulation using dCas9. These designed fusion RNAs have the capacity to both target promoter regions through their CRISPR sgRNA sequence as well as regulate promoter expression via multivalent recruitment of protein effectors on protein-binding motifs scaffolded on RNA origami structures. (**C**) Expression of varying sgRNAOs to regulate different genomic loci using dCas9 as a master regulator.

Here, we demonstrate the utilization of RNA origami structures as scaffolds carrying multiple functional motifs for dCas9-based regulation *in vivo* (Figure 1A). We create fusions of sgRNAs with computationally designed RNA origamis (termed sgRNAOs) and attempt to modulate the expression of genetic targets in *S. cerevisiae* yeast. The sgRNAOs have the capacity to bind to dCas9 and target specific loci while also carrying stable RNA motifs enabling additional functionality (Figure 1B). Using MS2 or PP7 aptamers as accessory motifs, we rationally design sgRNAOs capable of binding transcription factors that activate gene expression at a level directly related to the number of aptamer motifs. We then explore how regulatory performance is affected by the positioning of the protein-binding aptamers on the scaffold and the orientation of the RNA origami tile in regard to the sgRNA. Finally, we apply the sgRNAO system for the design of complex RNA programs controlling the violacein metabolic pathway and showcase its capacity to directly control the metabolic flux to enhance the violacein product yield.

## MATERIALS AND METHODS

### sgRNAO design

The RNA origamis were designed using the Revolvr software, which based on ViennaRNA package's minimum free energy (MFE) parameters generates RNA sequences that are predicted to have the desired folding characteristics (20,24). RNA motifs for the MS2 C5 variant and PP7 aptamers (25,26) and for the 3-way junction (UA_h_3WJ5) with a UA_handle motif (5′XU/AN_*n*_X3′) (27,28) were included as constrained sequences in the 2D blueprints (see RNA motifs in Supplementary Table S1. 2D blueprints of desired scaffold RNA structures were used to generate at least 30 sequences for each RNA origami design and promising candidates were selected based on their predicted folding efficiency, which was identified by low ‘ensemble diversity’ and ‘ensemble defect’ scores given by ViennaRNA and NUPACK, respectively. Final RNA origami sequences were then fused to sgRNAs following the CRISPR-Display INT method of internal fusion of lncRNAs to the engineered loop of sgRNAs (10). For certain designs, the sgRNA sequence was included in the starting 2D blueprints (see RNA blueprints and sequences in Supplementary Table S2).

### Strain creation

All engineered yeast strains were derived from the haploid *S. cerevisiae* strain Y02569 (BY4741; *MAT*a; *ura3*Δ*0*; *leu2*Δ*0*; *his3*Δ*1*; *met15*Δ*0*; YJR092w::*kanMX4*) provided by EUROSCARF. On all yeast strains, codon optimized *S. pyogenes* dCas9 is fused on the C-terminal with three tandem copies of SV40 nuclear localization signals (NLSs), while MCP-VP64 and PCP-VP64 are flanked with one N-terminal and one C-terminal copy of SV40 NLS. Open reading frames (ORFs) were taken from pJZC620 (a gift from Wendell Lim & Stanley Qi, Addgene plasmid #62282 ; http://n2t.net/addgene:62282; RRID:Addgene_62282) and sequences were adjusted for yeast MoClo toolkit compatibility (8,29). All sgRNA target sequences were derived from Zalatan *et al*, 2014 (8). For sgRNAO CRISPR characterization experiments, Y02569 was first transformed with the pGPY572 *LEU2* selection plasmid carrying dCas9 under the inducible LX promoter and LACI under the pPGK1 promoter thus creating the GPY572 strain (30). GPY572 was subsequently transformed with *URA3* selection plasmids carrying sgRNAO (or control RNAs), mVenus marker, and MCP/PCP expression cassettes (all plasmids shown in Supplementary Table S3). The 3MS2-SL sequence was taken from Shechner *et al.* and the scRNA-2xMS2 sequence from Zalatan *et al.* (8,10).

For violacein pathway strains, Y02569 was first transformed with pGPY634 (*LEU2* selection) carrying VioABCDE under constitutive expression (shown in Figure 4B) thus creating GPY634. GPY634 was subsequently transformed with the *URA3* plasmids pGPY698, pGPY702, pGPY703, pGPY712 and pGPY713 (Supplementary Table S4), carrying the dCas9, the corresponding sgRNA/sgRNAO (based on the genetic programs shown in Figure 5C and D) as well as the MCP-VP64 and PCP-VP64 expression cassettes, thus creating the GPY698, GPY702, GPY703, GPY712 and GPY713 strains, respectively. The negative control strain GPY700 was created by transforming the GPY634 with pGPY700, which only includes a dCas9 expression cassette. All constructs were integrated into the genome in single copies. All sgRNA/sgRNAOs are expressed by the SNR52 promoter with a SUP4 terminator.

*S. cerevisiae* yeast plasmids were assembled using Golden Gate based on the Yeast Toolkit for modular assembly developed by the Dueber lab and transformed in *E. coli* Turbo (NEB) cells (29). All biological part plasmids were ordered for synthesis from Integrated DNA technologies (IDT) or Twist Biosciences. *E. coli* selection was performed in lysogeny broth (LB) agar plates containing either 34 μg/ml chloramphenicol, 100 μg/ml carbenicillin or 50 μg/ml kanamycin. Yeast transformations were performed using a LiAc/ssDNA/PEG protocol (31). All yeast constructs were integrated into the genome in single copies. For yeast selection cells were cultured on synthetic drop-out glucose (SD-Glu) agar plates selecting for either uracil or leucine. For general proliferation, cells were cultured in synthetic complete glucose (SC-Glu) liquid media. LX promoter induction was performed in synthetic drop-out or synthetic complete galactose (SD-Gal) liquid media with IPTG. For flow cytometry experiments, cells were grown in SC-Glu or SC-Gal media with 2 mM IPTG for 24 h in liquid cultures at 30°C with shaking at 225  rpm. For violacein metabolic pathway compound extraction and HPLC analysis, cells were grown in either liquid (225 rpm) or solid SD-Glu cultures at 30 °C.

### Flow cytometry

Flow cytometry analysis used an ACEA NovoCyte 2100YR system. Signal coming from mVenus was detected using a 488 nm laser for excitation and a 530/30 nm band pass filter. Data was captured using NovoExpress software measuring 10 000 gated events based on forward and side scatter to isolate singlets. Representative flow cytometry traces shown in Supplementary Figure S1. Data was analyzed and graphs were created using GraphPad Prism.

### HPLC

Figure 5C strains were initially grown in SD Glu URA^−^LEU^−^ liquid cultures for 72 h at 30°C. For each strain, 2.5 ml of OD_600_ = 10 culture was then harvested and pelleted by centrifugation at 13 000 g. Supernatant was removed and cell pellet was resuspended in 500 μl methanol and lysed at 100°C for 20 min. Supernatant was then passed through a 0.22 μm filter before using it for HPLC analysis. Figure 5D strains were initially cultured on SD-Glu URA^−^LEU^−^ plates for 72 h at 30°C. Cells were then resuspended in liquid SD-Glu URA^−^LEU^−^ media and adjusted to OD_600_. 800 μl of OD_600_ = 8.3 culture was then pelleted by centrifugation at 13 000 g, and after supernatant removal pellet was resuspended in 200 μl methanol and lysed at 100°C for 20 min. Supernatant was passed through a 0.22 μm filter to remove cell debris. For HPLC measurements, either 200 μl (Figure 5C strains) or 100 μl (Figure 5D strains) of sample (50/50 of water + extract) were run on an Agilent 1200 Series LC system using an Agilent Extend-C18 column (150 × 4.6 mm, 3.5 μm). Solvent A (0.1% formic acid in water) and Solvent B (0.1% formic acid in acetonitrile) were used on the following method: Start at 5% Solvent B, 5% Solvent B for 2 min, transition to 98% Solvent B (9.3%/min), transition to 5% Solvent B (31%/min) and hold for 3 min (method adapted from Lee *et al.*, 2013) (32). Flow rate was 500 μl/min, column temperature at 30°C and absorbance was measured at 260 nm, 220 nm, 565 nm (shown in Figure 5C and D) and 600 nm using a UV/VIS detector. A violacein/deoxyviolacein mixed extract (Sigma-Aldrich) was used as a reference since pure standards were unavailable. Peak area calculations were performed using GraphPad Prism (shown in Supplementary Figure S2).

## RESULTS

### Expression of sgRNAOs enable CRISPR-mediated gene activation

**Figure 2.**
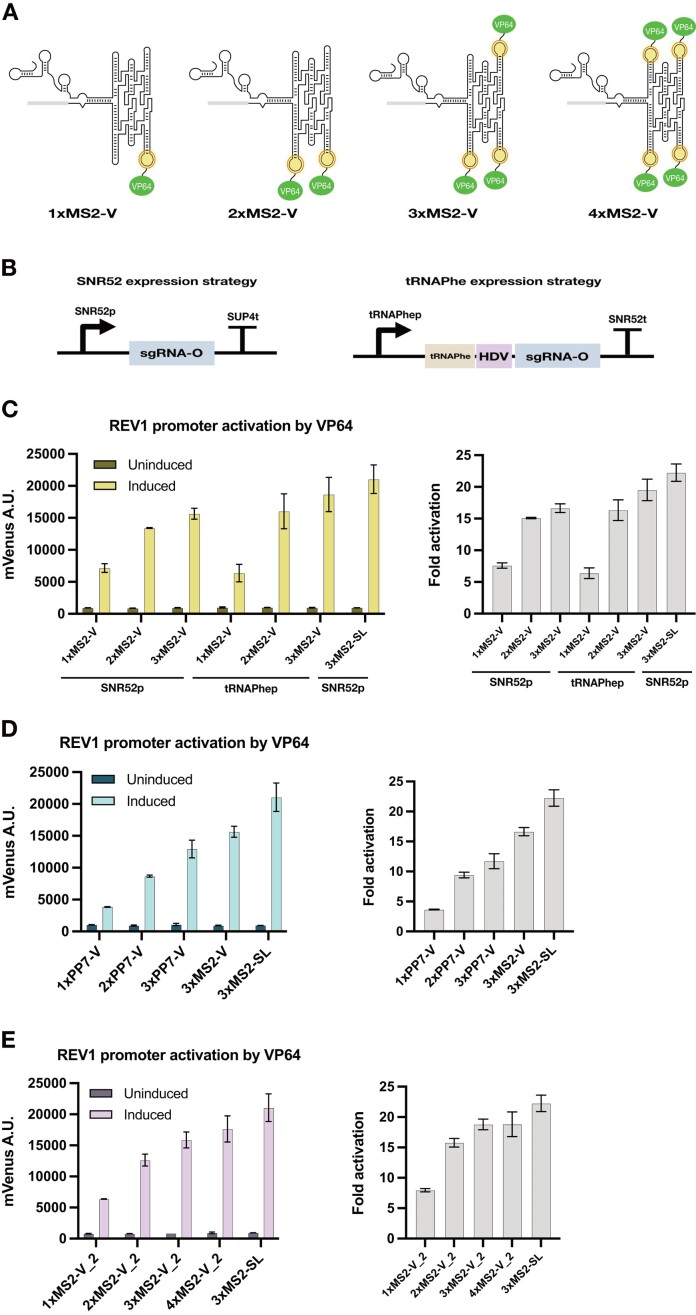
Utilizing RNA origami scaffolds for genomic regulation using CRISPR. (**A**) Secondary structure diagrams of 3-helix sgRNAOs carrying varying numbers of MS2 hairpins. (**B**) Expression strategies to ensure optimal transcription of long sgRNAOs. The SNR52 expression cassette constitutes of the SNR52 promoter and the SUP4 terminator. The tRNAPhe expression cassette constitutes of the tRNAPhe promoter, the SNR52 terminator and the sgRNAO flanked upstream by the tRNAPhe sequence and the HDV ribozyme sequence. (**C**) REV1 promoter activation by VP64 in strains expressing sgRNAOs upon induction of the CRISPR machinery verified by the expression of the mVenus fluorescence protein reporter in yeast. 3-helix sgRNAO constructs carrying up to three MS2 hairpins are shown. sgRNAOs are transcribed using either the SNR52 or tRNA Phe expression strategies. (**D**) REV1 promoter activation by VP64 in strains expressing sgRNAOs upon induction of the CRISPR machinery verified by the expression of the mVenus fluorescence protein reporter in yeast. 3-helix sgRNAO constructs carrying varying numbers of MS2 or PP7 hairpins are shown. All sgRNAOs are expressed from the SNR52 promoter. (**E**) REV1 promoter activation by VP64 in strains expressing sgRNAOs upon induction of the CRISPR machinery verified by the expression of the mVenus fluorescence protein reporter in yeast. 3-helix sgRNAO constructs sharing the same base RNA origami scaffold sequence carrying up to four MS2 hairpins are shown. All sgRNAOs are expressed from the SNR52 promoter. In all panels, a 3xMS2-sgRNA control (3xMS2-SL) obtained by Shechner *et al.* is included. Fluorescence data from mVenus expression was obtained by flow cytometry before and after dCas9 induction in strains expressing sgRNAOs targeting the REV1 promoter. Mean values and ± SD from biological triplicates are shown. Calculated relative fold activation ± SEM and after induction is also provided.

We first incorporated large computationally designed RNA origami scaffolds carrying protein-binding aptamers to sgRNAs (Figure 1A) thus creating sgRNA-RNA origami (sgRNAO) fusions capable of gene regulation in *S. cerevisiae* yeast (Figure 1B). This allows for the expression of varying sgRNAO designs that target different genetic loci (Figure 1C). Initial designs consisted of 3-helix (3H) RNA origamis, carrying up to three MS2 coat protein-binding aptamer motifs, fused in a vertical (V) orientation to the engineered stem loop drawn horizontally (termed MS2-V, see Figure 2A, see Supplementary Table S2 for blueprints) (33). The sgRNA engineered loop has formerly been shown to enable incorporation of structurally diverse RNA domains and allow for stronger gene activation (10).

To identify how to best express the sgRNAOs, we attempted two different strategies (Figure 2B). In yeast CRISPR/(d)Cas9 applications, a popular mode of sgRNA expression is by using the robust SNR52 promoter (34). Although expression of sgRNAs from the SNR52 promoter is the most common, Pol III promoters usually produce relatively short transcripts thus limiting the maximum length of lncRNAs allowed (10). To avoid the production of truncated transcripts, lengths of sgRNAOs were initially kept to a minimum (∼450 nt) through the implementation of the aforementioned 3-helix design. Moreover, in addition to the SNR52 promoter expression strategy, sgRNAOs carrying up to three MS2 motifs were also expressed using a tRNA expression strategy. In this case, sgRNAOs were placed downstream of the phenylalanine tRNA (tRNA^Phe^) and its Pol III promoter (tRNA^Phe^p) sequence, separated by the self-cleaving hepatitis delta virus (HDV) ribozyme sequence. This strategy, which enables the use of native host promoters, has been shown to lead to improved sgRNA stability, while the HDV ribozyme ensures uncoupling from the translation machinery (35).

The initial goal was to verify the capacity of sgRNAOs to properly express, fold and mediate their CRISPR-activation functions *in vivo*. For this, sgRNAO designs carrying MS2 binding aptamers, capable of guiding dCas9 to a target location and recruiting the VP64 (Virion protein 64, fusion of 4xVP16) transcriptional activation protein domain fused to the MS2 coat protein domain, were implemented (36). *S. cerevisiae* strains were created, carrying the dCas9 gene under a galactose and IPTG-inducible promoter (LX promoter described by Ellis *et al*), an mVenus reporter expression cassette under the very weak REV1 promoter (REV1p), sgRNAO expression cassettes following either the SNR52p or the tRNA^Phe^p expression strategies and MCP-VP64 expression cassettes under constitutive TDH3 promoter (TDH3p) expression (30). REV1p activation by VP64 using 3-helix sgRNAOs carrying up to three MS2 aptamers as well as a control sgRNA carrying an internal fusion of three tandem MS2 repeats (3xMS2-SL) is shown in Figure 2C (10). Relative fold activation of REV1p after induction for all strains is also included. Results confirm sgRNAO-mediated activation of REV1p for all MS2 strains and a stepwise increase in expression following the number of protein-binding aptamers. Expression of sgRNAOs from tRNA^Phe^p leads to 19% higher mean REV1p activation and 17% higher fold activation for the 3xMS2-V when compared to SNR52p but shows less consistency between samples. The 3xMS2-SL control exhibits 35% higher activation ability than the 3xMS2-V when under the SNR52p but is also more variable between samples. Both the 2xMS2-V and 3xMS2-V sgRNAOs exhibit higher mean activation compared to the 2xMS2-scRNA (52% and 78%, respectively, Supplementary Figure S3). Overall, the SNR52p expression strategy of sgRNAOs allows for a consistent transcriptional activation profile, with the 3xMS2-V sgRNAO performing on a level close to the 3xMS2-SL.

### PP7 functions as an alternative binding motif for sgRNAOs

One of the advantages of RNA origami scaffolds, is their compatibility with different functional motifs. To expand the genetic regulation options provided by RNA origami scaffolds and as an extension to sgRNAOs, the PP7 binding aptamer was also explored (26). Similar to their MS2 counterparts, 3-helix sgRNAOs carrying up to three PP7 RNA motifs were assessed for their ability to upregulate REV1p through recruitment of PCP-VP64 (Figure 2D). Similar to MS2 carrying sgRNAO, a stepwise increase was observed between 1xPP7-V, 2xPP7-V and 3xPP7-V. Overall REV1p activation from 3xPP7-V is 17% lower than the one of 3xMS2-V, potentially explained by the reduced protein-binding affinity of PP7 in comparison to MS2 (26,37). In addition, average fold-activation of 3xPP7-V is 30% less than the one of 3xMS2-V (Figure 2D). On average, PP7-V constructs exhibit ∼33% lower activation when compared to MS2-V constructs (also see Supplementary Figure S3), a pattern also observed using scRNAs (8). Overall, sgRNAOs carrying PP7 aptamers are able to upregulate genes in the presence of dCas9, and similar to the MS2-V sgRNAO constructs, provide a stepwise increase in expression depending on the number of binding aptamers.

### Generating sgRNAO derivatives from a 4xMS2 RNA origami

Initial sgRNAOs were created using unique RNA origami scaffold sequences for each design. To explore the flexibility of the RNA origami scaffolds at accommodating accessory RNA motifs, an alternative design process was attempted in the form of sgRNAOs that derive from a common sequence. An sgRNAO carrying four MS2 aptamers (4xMS2-V_2) was generated and one, two and three-MS2 aptamer sgRNAO derivatives (1xMS2-V_2, 2xMS2-V_2, 3xMS2-V_2) were created by replacing MS2 aptamers with tetraloop motifs. Figure 2E shows the regulatory performance of the four variants. An increase in REV1 promoter activation following the number of protein-binding aptamers is observed once again for up to three MS2 motifs. A minimal benefit in mean activation is observed when the number of aptamers is increased to four (11% increase over 3xMS2-V_2) which is further verified by fold-activation calculations (no increase over 3xMS2-V_2) shown in Figure 2E. This effect might indicate higher 4xMS2-V_2 misfolding or saturation of the system.

To test whether our system is limited by the availability of MCP-VP64 ligands, in connection with an increasing number of MS2 aptamers on sgRNAOs which could explain 4xMS2-V_2’s performance, we attempted to express MCP-VP64 at either significantly lower or higher levels (Supplementary Figure S4). Increasing the number of protein binding motifs on the RNA origami scaffolds essentially increases the ligand level requirements for scaffold/ligand stoichiometry and this could theoretically lead to even a decrease in function when all other RNA and protein expression characteristics remain the same (38,39). The stepwise increase pattern between 1xMS2, 2xMS2 and 3xMS2 sgRNAOs variants was similar, which indicates that the system is not limited by ligand availability (Supplementary Figure S4).

### Aptamer positioning on scaffolds affects transcriptional activation

**Figure 3.**
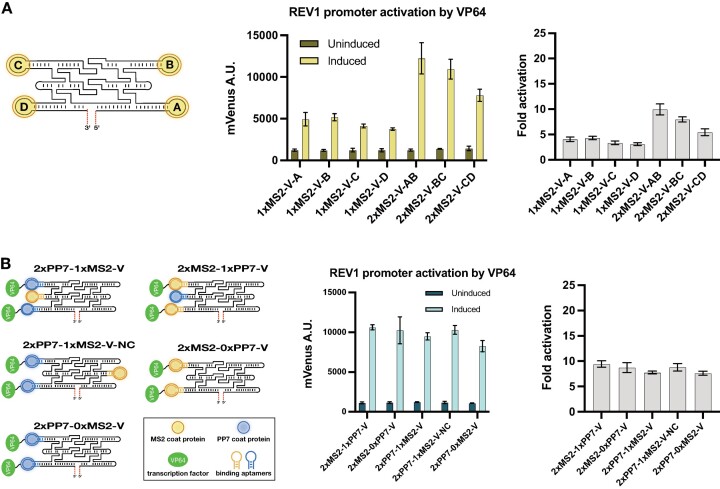
Effects of aptamer positioning on 3-helix sgRNAO. (**A**) Characterization of four protein binding aptamer positions on 3-helix sgRNAOs for their capacity to regulate mVenus gene expression. 3-helix sgRNAO constructs carrying up to two MS2 hairpins placed on different scaffolding positions are shown. (**B**) Assessment of additional protein-binding aptamer positions and their impact on mVenus gene regulation. 3-helix sgRNAO constructs carrying MS2 and/or PP7 hairpins placed on all three RNA origami helixes are shown. Fluorescence data from mVenus expression was obtained by flow cytometry before and after dCas9 induction in strains expressing sgRNAOs targeting the REV1 promoter. Mean values and ± SD from biological triplicates are shown. Calculated relative fold activation ± SEM and after induction is also provided.

Following the demonstration of sgRNAOs’ ability to carry multiple RNA protein-binding motifs and regulate expression, we opted to characterize the potential protein-binding aptamer positions of the 3-helix sgRNAO designs independently and identify which positions offer the best regulatory performance. Initially, we focused on the four positions used on the 4xMS2-V_2 sgRNAO (A, B, C and D positions on the first and third helix of a 3-helix RNA origami as shown in Figure 3A). Four 1xMS2 and three 2xMS2 sgRNAO variants were tested for their ability to recruit MCP-VP64 and activate REV1 promoter expression (Figure 3A). Between the 1xMS2-V sgRNAO variants, the 1xMS2-V-B sgRNAO exhibits the highest REV1 promoter activation; 5% over 1xMS2-V-A, 25% over 1xMS2-V-C and 38% over 1xMS2-D. The 2xMS2-V sgRNAO variants, follow an additive pattern that corresponds to the activation levels of the 1xMS2-V sgRNAO variants, with the 2xMS2-V-AB exhibiting 12% and 57% higher REV1 promoter activation than the 2xMS2-V-BC and 2xMS2-V-CD, respectively. These results indicate that placing aptamers on positions A and B lead to higher transcriptional activation than on positions C and D, with D offering the lowest activation. It appears that positions closer to the 5′ end of the RNA origami, and thus the sgRNAO, offer better regulatory performance which might be attributed to differences in sgRNAO folding efficiencies or spatial positioning between MCP-VP64 motifs and DNA target. The decreased regulatory impact of positions C and D might also explain, at least partially, the diminishing benefits of increasing protein-binding aptamers placed on sgRNAOs as shown in Figure 2.

### Expanding the number of potential protein-binding sites

After characterizing placement of protein-binding aptamers on the first and third helixes of a 3-helix sgRNAO, we attempted to also test the possibility of protein recruitment on the middle helix since it has been formerly observed that protein recruitment on adjacent helices may inhibit binding (20). We designed sgRNAOs carrying MS2 and PP7 motifs on all three helixes, recruiting MCP and PCP dimers respectively (shown in Figure 3B). To test for potential hindrance caused by the binding of MCP and PCP dimers close to each other and its effect on overall gene regulation, sgRNAOs carrying three binding aptamers on the same side of a 3-helix RNA origami tile were generated, with the middle helix always carrying a different binding aptamer from the other two and recruiting a binding protein that is not fused to VP64. Figure 3B shows the regulatory performance of the 2xPP7-1xMS2-V (recruiting PCP-VP64 and MCP) and 2xMS2-1xPP7-V (recruiting MCP-VP64 and PCP) sgRNAOs carrying binding aptamers positioned on the same side of the tile as well as the 2xPP7-1xMS2-V-NC and 2xMS2-1xPP7-V-NC sgRNAO where the non-regulating protein-binding motif is positioned on the opposite side. A 2xPP7-0xMS2-V control sgRNAO lacking any protein-binding motif on the middle helix is also included. Results show that recruitment of binding proteins on the middle helix doesn’t hinder protein binding on other adjacent positions as indicated by the similar transcriptional activation capacity of the sgRNAOs tested. These results further underline the RNA origami's ability to provide a stable scaffold with appropriate spacing and indicate that 3-helix sgRNAOs carrying up to six protein-binding aptamers is an option and assuming VP64 protein size allows it, could enable the recruitment of up to six MCP-VP64 dimers.

### Effects of RNA origami orientation and size on sgRNA function

**Figure 4.**
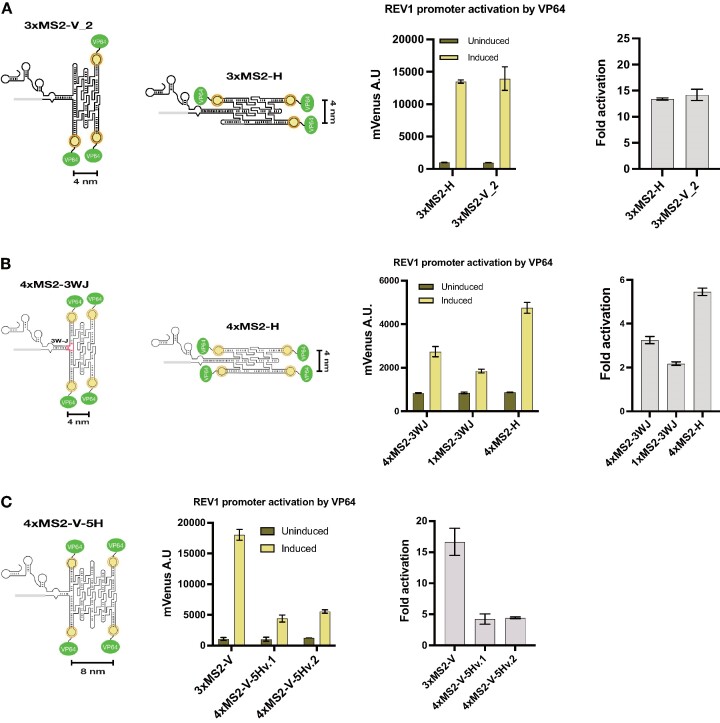
Effects of RNA origami orientation and size on sgRNA function. (**A**) Characterization of RNA origami—sgRNA fusion configurations for their capacity to regulate mVenus gene expression. 3-helix sgRNAO constructs carrying three MS2 hairpins are shown. (**B**) REV1 promoter activation by VP64 in strains expressing sgRNAOs verified by the activation of the mVenus gene reporter in yeast. sgRNAOs consist of 3-helix RNA origami sequences carrying varying numbers of MS2 binding aptamers fused to the sgRNA in a horizontal (4xMS2-H-3H) or vertical (4xMS2-3WJ-3H and 1xMS2-3WJ-3H) orientation. sgRNAOs are transcribed using the SNR52 promoter expression strategy. (**C**) REV1 promoter activation by VP64 in strains expressing sgRNAOs verified by the activation of the mVenus gene reporter in yeast. sgRNAOs consist of 3-helix or 5-helix RNA origami sequences carrying varying numbers of MS2 binding aptamers (3xMS2-V-3H, 4xMS2-V-5H version 1 and version 2). sgRNAOs are transcribed using the tRNA-Phe promoter expression strategy. Fluorescence data from mVenus expression was obtained by flow cytometry before and after dCas9 induction in strains expressing sgRNAOs targeting the REV1 promoter. Mean values and ± SD from biological triplicates are shown apart from 4xMS2-V-5Hv.2 which is shown in duplicates. Calculated relative fold activation ± SEM and after induction is also provided.

To explore the design space of the sgRNAO system, we investigated whether specific structural modifications on the RNA origami tiles and specific fusion types to the sgRNA sequence can affect sgRNAO-mediated gene regulation. We first opted to alter the orientation of the RNA origami scaffolds in regard to the sgRNA sequence as well as the overall CRISPR complex. A 3xMS2 sgRNAO was designed that carries RNA origami scaffolds fused to the sgRNA horizontal (H) to the engineered loop (3xMS2-H, Figure 4A). The RNA origami is fused after extending the engineered sgRNA stem by 20 bp to create additional space for MCP-VP64 to bind. Figure 4A shows REV1p activation by VP64 for strains expressing 3xMS2-V_2 and 3xMS2-H sgRNAOs. Overall, REV1 expression and fold-activation is similar between strains underlining the viability of both fusion schemes which could indicate that the actual 3D arrangement of the RNA origami scaffold between the two schemes is similar. This similarity may be explained by modeling the three-way junction between the RNA origami scaffold and the engineered stem of 3xMS2-V_2 as a family B junction which would orient the vertical origami in a configuration close to the horizontal orientation (40).

Following this, to increase confidence in orientational differences between fused RNA origamis on sgRNAOs, two new 4xMS2 and one 1xMS2 sgRNAOs were designed that carry RNA origami scaffolds fused to the sgRNA either vertical in relation to the engineered loop using a family A three-way junction (3WJ) motif (4xMS2-3WJ) or horizontal to the engineered loop (4xMS2-H, Figure 4B) (40). In the 4xMS2-3WJ, the RNA origami is fused to the sgRNA using a 3WJ expected to provide a more constrained T-shape of the connection between the RNA origami scaffold and the CRISPR complex (27,28). For the 4xMS2-H, the RNA origami is fused horizontally after extending the engineered sgRNA stem by 20 bp to create additional space for MCP-VP64 to bind. Figure 4B shows REV1p activation by VP64 for strains expressing 4xMS2-3WJ, 1xMS2-3WJ and 4xMS2-H sgRNAOs. Overall, REV1p expression and fold activation is lower than the MS2-V sgRNAOs shown in Figure 2. The 3WJ and H sgRNAOs were designed with the sgRNA sequence incorporated into the blueprints which could explain their poor performance. The best performant sgRNAO was the 4xMS2-H which exhibited 73% higher activation than 4xMS2-3WJ, which might indicate that the positioning of the RNA scaffold has an effect on promoter activation but we cannot rule out misfolding as a possible explanation.

To investigate the compatibility of our sgRNAO system with even larger RNA origamis, we designed 5-helix RNA origamis carrying four MS2 aptamers (general schematic shown in Figure 4C). Expression was performed following the tRNA^Phe^p strategy since it has shown to lead to slightly higher REV1p activation in previous experiments. Two sgRNAO sequences (4xMS2-V-5Hv.1 and 4xMS2-V-5Hv.2) were generated and compared with the 3xMS2-V (Figure 4C). When compared to 3xMS2-V under tRNA^Phe^p expression, the 4xMS2-V-5Hv.1 and 4xMS2-V-5Hv.2 exhibited 24% and 31% of mean mVenus fluorescence, respectively. This could be attributed to limitations of tRNA^Phe^p in generating transcripts of increased length or lower folding efficiency compared to 3-helix variants.

### sgRNAOs redirect metabolic flux of the Violacein pathway

**Figure 5.**
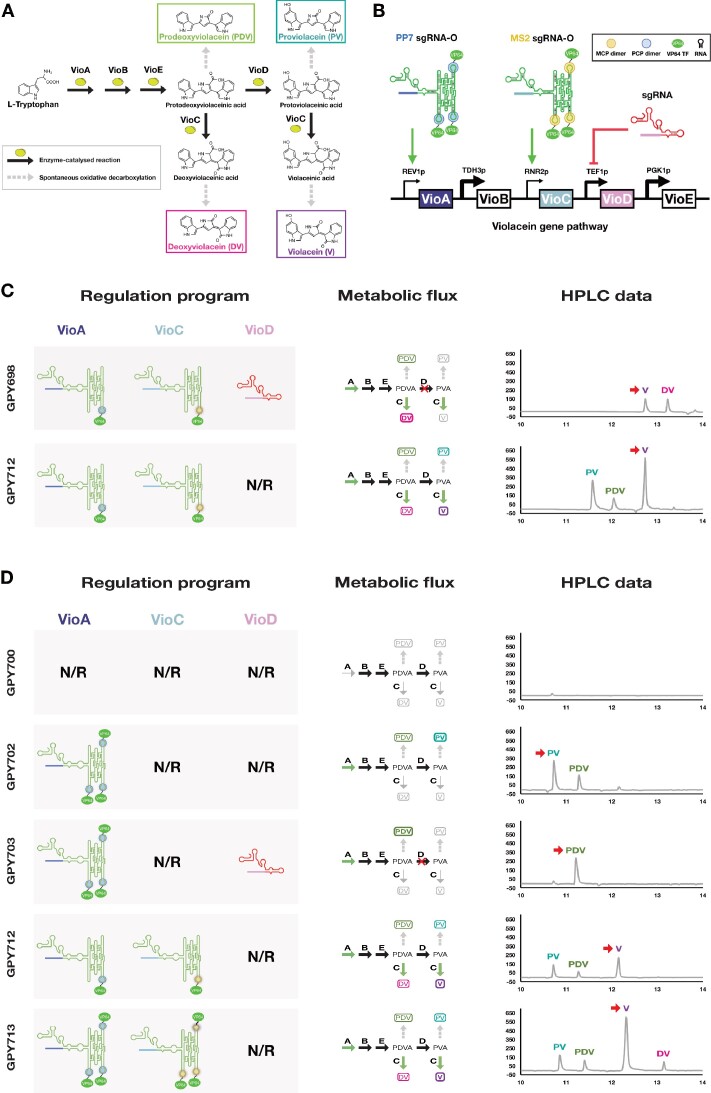
Implementing sgRNAO-driven CRISPR regulatory programs for the control of the violacein metabolic pathway. (**A**) The violacein biosynthesis in bacteria. Through a combination of five enzymatic and one non-enzymatic steps L-Trp is converted to violacein. In addition to violacein, the branched pathway can lead to the synthesis of three additional compounds through steps catalysed by VioC and VioD. (**B**) Transferring the five violacein pathway enzymes to *S. cerevisiae* for sgRNAO-driven CRISPR regulation. Out of the five enzymes, VioB and VioE are constitutively expressed by strong promoters (TDH3 and PGK1, respectively), VioD by an average strength promoter (TEF1), and VioA and VioC by very weak promoters (REV1 and RNR2, respectively). At its default state, this genetic pathway is unable to lead to the biosynthesis of significant amounts of any of the four possible compounds due to the very low expression of VioA. Utilizing a combination of sgRNAOs and sgRNAs targeting VioA, VioC and VioD, RNA programs that lead to the production of all four compounds can be achieved. (**C**) HPLC analysis of violacein pathway product distribution from *S. cerevisiae* strains expressing varying RNA programs. Cells were grown in SC (synthetic complete) glucose media for 72 h and compounds were extracted in methanol. (**D**) HPLC analysis of violacein pathway product distribution from *S. cerevisiae* strains expressing varying RNA programs. Cells were grown in SD-URA-LEU glucose solid cultures for 72 h and compounds were extracted in methanol. N/R: No regulation. Red arrows are pointing to dominant compounds based on peak area calculations (shown in Supplementary Figure S2).

Next, we applied the sgRNAO technology in the context of complex transcriptional programs for the control of the branched violacein metabolic pathway and attempt to shift predominant production to any of the four distinctly colored products of the pathway (Figure 5A). This work extends the study performed on creating complex synthetic regulatory programs using the scRNA technology (8). The violacein pathway consists of five enzymes (VioA, VioB, VioC, VioD and VioE) catalyzing the biosynthesis of violacein (V) from L-Tryptophan. In addition, regulation of enzymes VioC and VioD can also lead to production of prodeoxyviolacein (PDV), deoxyviolacein (DV) and proviolacein (PV). For our sgRNAO experiments, several yeast strains carrying different dCas9-CRISPR transcriptional programs were tested and assessed for violacein pathway metabolite production. VioA, VioC and VioD were the genetic CRISPR targets, regulated by a combination of activating sgRNAOs and an inhibiting sgRNA (through dCas9 mediated CRISPR interference) (3). Strains carried the complete violacein multi-gene pathway (Figure 5B) as well as the elements of the dCas9-CRISPR machinery under constitutive expression. As a proof-of-concept experiment, sgRNAOs based both on MS2 and PP7 binding motifs were used. VioA was placed under the control of the very weak REV1 promoter (REV1p) while VioC was placed under the control of the very weak RNR2 promoter (RNR2p), targeted by PP7-carrying and MS2-carrying sgRNAOs, respectively. VioD was placed under the control of the medium-strength TEF1 promoter which was targeted by an sgRNA to induce CRIPSRi (3). Initially, we attempted to verify whether sgRNAO-mediated production of V as the dominant product can be achieved through VioA and VioC upregulation and a yeast strain carrying the necessary RNA program was created (GPY712). In addition, a second strain (GPY698) targeting the production of DV through upregulation of VioA and VioC with parallel inhibition of VioD. Based on past work, production of DV as a dominant product has shown to be problematic, assumingly due to VioC’s inefficiency to convert prodeoxyviolaceinic acid (PDVA) to DV (8). Both strains carry sgRNAOs consisting of 3-helix RNA origami tiles carrying one copy of either an MS2 or a PP7 aptamer (1xMS2-V targeting RNR2p or 1xPP7-V targeting REV1p, respectively). HPLC data suggests that both GPY698 and GPY712 strains grown in liquid cultures are capable to execute their intended regulatory programs and lead to production of DV and V as dominant products, respectively (Figure 5C and Supplementary Figure S2). However, DV production from GPY698 was low, while V was also present at slightly lower levels. More importantly, contrary to past work there appears to be no PDV production which in combination with some level of V production suggests incomplete repression of VioD and low upregulation of VioC from an sgRNAO carrying a single MS2 aptamer. On the other hand, GPY712 clearly leads to production of V as the dominant product, while smaller amounts of PDV and PV are also present, as a result of their nonenzymatic conversion from PDVA and protoviolaceininc acid (PVA), respectively. Finally, relative production of V from GPY712 is 3.8x higher than the production of DV from GPY698, further implying VioC’s reduced capacity to convert PDVA to DV.

### Increased number of sgRNAO protein binding sites enhances metabolic flux

After showing that sgRNAOs can be successfully used to create regulatory RNA programs and create strains capable of producing V and DV as the dominant products of the violacein pathway, we tested RNA programs targeting production of the remaining two products of the violacein pathway (PV and PDV) as well as enhance overall production. To achieve this, three new strains were created based on sgRNAOs carrying three copies of either the MS2 or the PP7 aptamers (3xMS2-V targeting RNR2p and 3xPP7-V targeting REV1p); GPY702 targeting PV production, GPY703 targeting PDV production and GPY713 targeting V production. Figure 5D and Supplementary Figure S2 show the HPLC analysis of GPY702, GPY703 and GPY713 along with the violacein producing strain GPY712 that only carries sgRNAOs with single protein binding motifs. A negative control strain carrying only the VioABCDE pathway and expressing dCas9 was also included to verify the inability of VioA to initiate L-tryptophan conversion when a very weak promoter is used. For this set of experiments, all strains were cultured on solid media. Data showed that all three new strains could redirect metabolic flux to their intended targets. GPY702 produced the intended PV as the dominant product, while PDV was also produced through non-enzymatic conversion of PDVA. GPY703 produced almost exclusively PDV as intended. GPY713, following a pattern similar to GPY712 produced V as the dominant product while all other three compounds were also produced but at significantly lower levels. Most importantly, GPY713, expressing sgRNAOs with three copies of MS2 and PP7 aptamers, could produce 2.7x higher relative amounts of V compared to GPY712, expressing sgRNAOs carrying one copy of aptamers.

## DISCUSSION

The aim of this study is to verify that RNA origami can work as a scaffold *in vivo*, demonstrating the incorporation of different functional elements. Here, utilizing the RNA origami scaffold technology previously described in *in vitro* experiments, we opt to create complex RNAs compatible with CRISPR activation thus proposing a powerful and modular molecular platform for synthetic biology applications. Fusions of sgRNA sequences targeting several genomic promoter regions with RNA origami-based scaffolds, recruiting several activating domains, are applied for transcriptional regulation. To achieve this, specific RNA design rules are applied, also considering host-related expression limitations. The significance of this method is further validated by the creation of complex RNA programs for the control of a branched metabolic pathway.

Here, we have showcased that RNA origamis carrying protein-binding aptamers can be expressed in eukaryotes and successfully fold to enable transcriptional activation. Since sgRNAOs have to localize in the nucleus and scaffold RNA sequences can often be quite long, this imposed certain design limitations when attempting to express them from Pol III promoters. Therefore, sgRNAO sizes were kept to a minimum for the purposes of this study. Expression of sgRNAs from sequence-length resilient Pol II promoters has been shown in the past and several strategies to avoid downstream processing and ensure nuclear localization have been deployed, including the incorporation of self-cleaving ribozymes (41,42). These approaches are generally less robust than expression from Pol III promoters and might lead to products prone to hydrolysis and thus another strategy for expression and nuclear localization of lncRNAs which mimics small nuclear RNA (snRNA) biogenesis has been described in mammalian cells using internal U1 and U2 elements which are unfortunately not well characterized in yeast (10). Here, we focused exclusively on Pol III expression strategies which for the goals of this study were proven adequate. It is important to note that since the dCas9-binding sequence of the sgRNAO is positioned on the very 3′ end of the transcript, successful dCas9 binding also ensures the presence of the complete RNA origami scaffold.

To exhibit compatibility of the system with different motifs, both the MS2 and the PP7 aptamers were selected and assessed within the CRISPR activation context. Scaffolding of multiple aptamers for multivalent recruitment of proteins was exhibited using sgRNAOs carrying up to four aptamers. The MS2 and PP7 aptamers have previously been shown to be orthogonal to each other even within the context of RNA scaffold-mediated CRISPR activation (8). From our results, it is evident that there is a stepwise increase in promoter activation for up to three scaffolded aptamers and that MS2 generally leads to higher fold activation which is consistent to previous works and the reported binding affinities for these aptamers (26,37). Expanding the number of scaffolded aptamers to four, only marginally increased activation which could be attributed to scaffold/aptamer misfolding, larger number of truncated transcripts due the increased length of these sgRNAOs or reaching a natural threshold for VP64-mediated transcriptional activation. In general, VP64 allows for very specific gene regulation when incorporated into a CRISPR-dCas9 system but also has a more limited activating potential compared to other alternatives (43).

Spatial distancing dictated by RNA aptamer positioning and scaffold orientation appears to have an effect on promoter activation. This has also been previously demonstrated for scaffolded enzymes where small perturbations of a scaffold affect enzymatic activity (17). Our data suggested weaker performance of certain scaffolding positions that could explain the diminishing returns of increasing aptamer numbers either due to RNA area-specific misfolding or three-dimensional interference of the CRISPR machinery to the protein-binding aptamers. Three-dimensional spacing of aptamers can also be controlled by overall scaffold orientation. Our results indicate that there is an effect on transcriptional activation by certain sgRNAO designs but this needs to be further explored.

The design of sgRNAOs is dictated by multiple goals including its transcriptional efficiency within the cell, proper structure folding and optimal placement of the protein-binding motifs. Internal fusion of RNA scaffold sequence to the sgRNA, not only appears to increase stability of the overall structure but can also place scaffolded proteins closer to the promoter sequence potentially increasing regulation. Furthermore, internal fusion of RNA scaffolds essentially places the tracer RNA sequence of the sgRNA on the 3′ end of the overall sequence thus ensuring that only complete transcripts are capable of dCas9 binding. As a result, all transcripts that reach their genomic targets should be complete sgRNAOs and this also acts as a control of Pol III transcriptional capacity.

Overall, this work aims to set the foundation for the expression of large RNA origami scaffolds *in vivo* for synthetic biology applications. RNA origami is highly modular and should allow the incorporation of other high-tier regulatory elements, such as RNA switches, enabling the creation of complex regulatory programs from single RNA scaffolds thus minimizing the number of RNA or other biological elements involved. With RNA being a simpler molecule to design and express *in vivo* than proteins, as well as imposing less burden, sgRNAOs can act as an important tool for synthetic biology and metabolic engineering.

## DATA AVAILABILITY

Data and materials are available upon request from the corresponding author.

## Supplementary Material

gkac470_Supplemental_File
