# Supplementary material for: Utilizing RNA origami scaffolds in Saccharomyces cerevisiae for dCas9-mediated transcriptional control
Source: Nucleic Acids Res. 2022 Jun 1;50(12):7176–87. doi: 10.1093/nar/gkac470 (PMC9262615; doi:10.1093/nar/gkac470)
Supplement: gkac470_Supplemental_File [file gkac470_supplemental_file.pdf]

Supplementary Data

"Utilizing RNA origami scaffolds in *Saccharomyces cerevisiae* for dCas9-mediated transcriptional control"

Georgios Pothoulakis, Michael TG. Nguyen, Ebbe S. Andersen

Table of Contents

SUPPLEMENTARY FIGURES 2

SUPPLEMENTARY FIGURE 1. REPRESENTATIVE FLOW CYTOMETRY TRACES. 2
SUPPLEMENTARY FIGURE 2. HPLC ANALYSIS OF VIOLACEIN PATHWAY RELATED CONSTRUCTS. 3
SUPPLEMENTARY FIGURE 3. COMPARISON OF SGRNAOS WITH PREVIOUSLY REPORTED DESIGNS. 4
SUPPLEMENTARY FIGURE 4. REV1 PROMOTER ACTIVATION WHEN VARYING MCP-VP64 EXPRESSION. 5

RNA BLUEPRINTS AND SEQUENCES 6

SUPPLEMENTARY TABLE 1. MS2, PP7 AND 3WJ MOTIFS USED IN THIS STUDY. 6
SUPPLEMENTARY TABLE 2. RNA SEQUENCES AND 2D BLUEPRINTS OF RNA ORIGAMI TILES AND SGRNAOS. 7

PLASMIDS 17

SUPPLEMENTARY TABLE 3. YEAST PLASMIDS CREATED FOR SGRNA-O MEDIATED REV1 UPREGULATION. 17
SUPPLEMENTARY TABLE 4. YEAST PLASMIDS CREATED FOR VIOLACEIN PATHWAY REGULATION. 19

Supplementary Figures

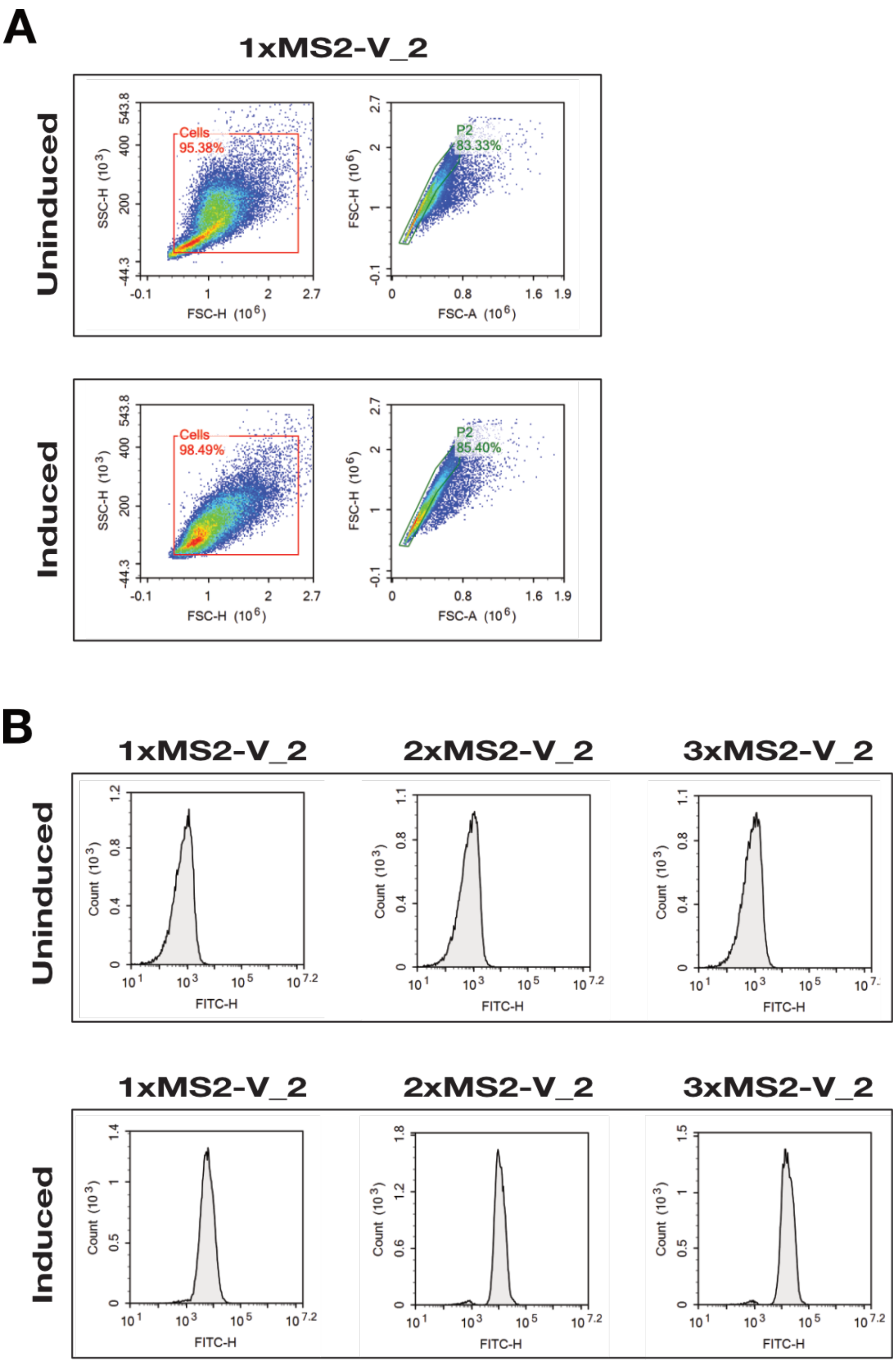

Supplementary Figure 1. Representative Flow cytometry traces.
(A) Sample gating procedure. Representative scatter plots of flow cytometry events collected for a strain expressing 1xMS2-V_2 sgRNAO. Events are first gated based on the forward (FSC-H) and side (FSC-A) scatters to exclude non-yeast events. Yeast events (events inside the "Cells" population) are further gated to isolate singlets ("P2" population) **(B)** Representative histograms showing cell distribution on the FITC-H (530/30 nm) channel for cells expressing 1xMS2-V_2. 2xMS2-V_2 and 3xMS2-V_2. All events are contained within the "P2" population shown in A.
Graphs were generated using NovoExpress software.

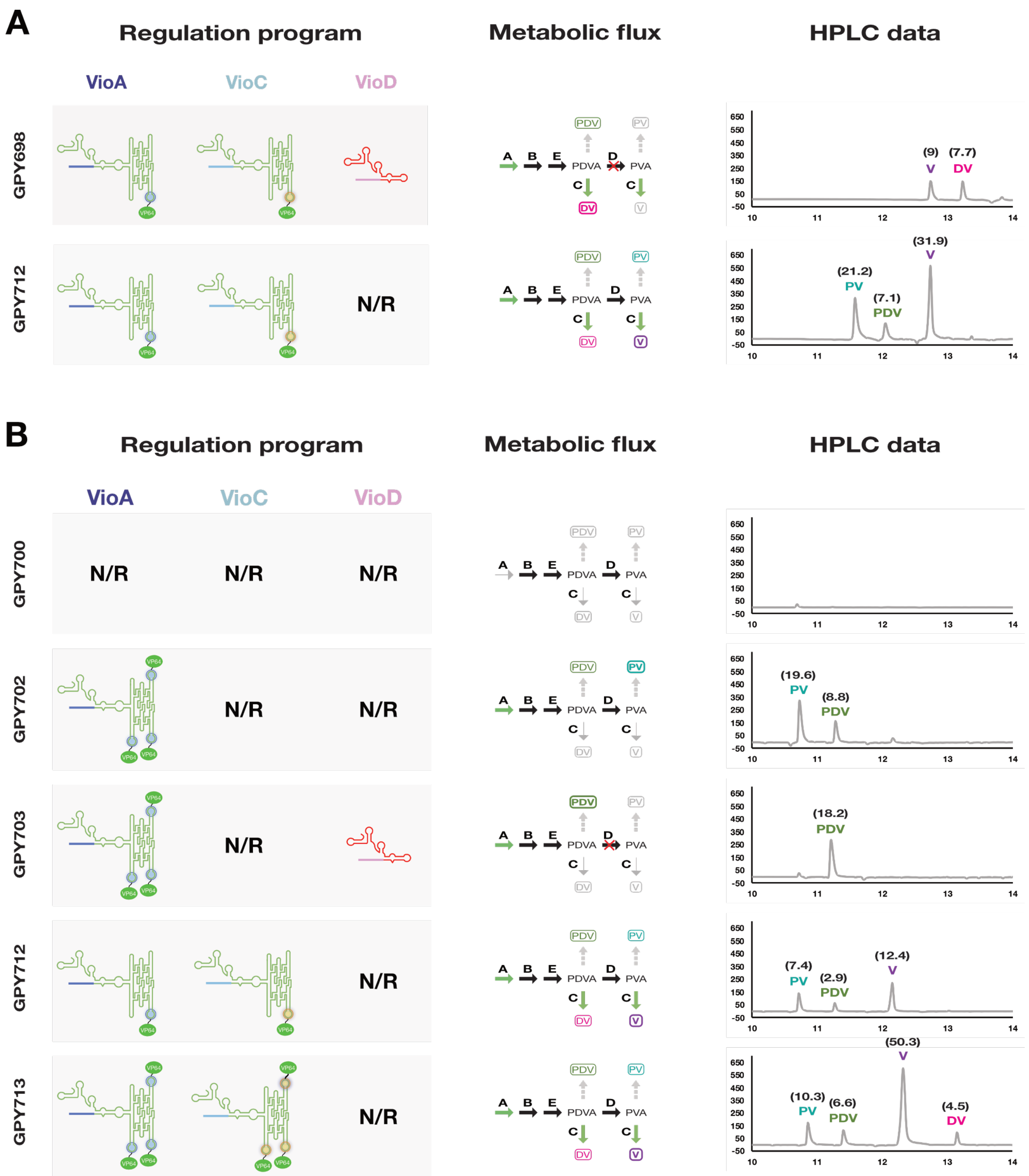

Supplementary Figure 2. HPLC analysis of Violacein pathway related constructs. **(A)** HPLC analysis of violacein pathway product distribution from *S. cerevisiae* strains expressing varying RNA programs. Cells were grown in SC (synthetic complete) glucose media for 72 h and compounds were extracted in methanol. **(B)** HPLC analysis of violacein pathway product distribution from *S. cerevisiae* strains expressing varying RNA programs. Cells were grown in SD-URA-LEU glucose solid cultures for 72 h and compounds were extracted in methanol. N/R: No regulation. Peak area values are shown in brackets above every major peak.

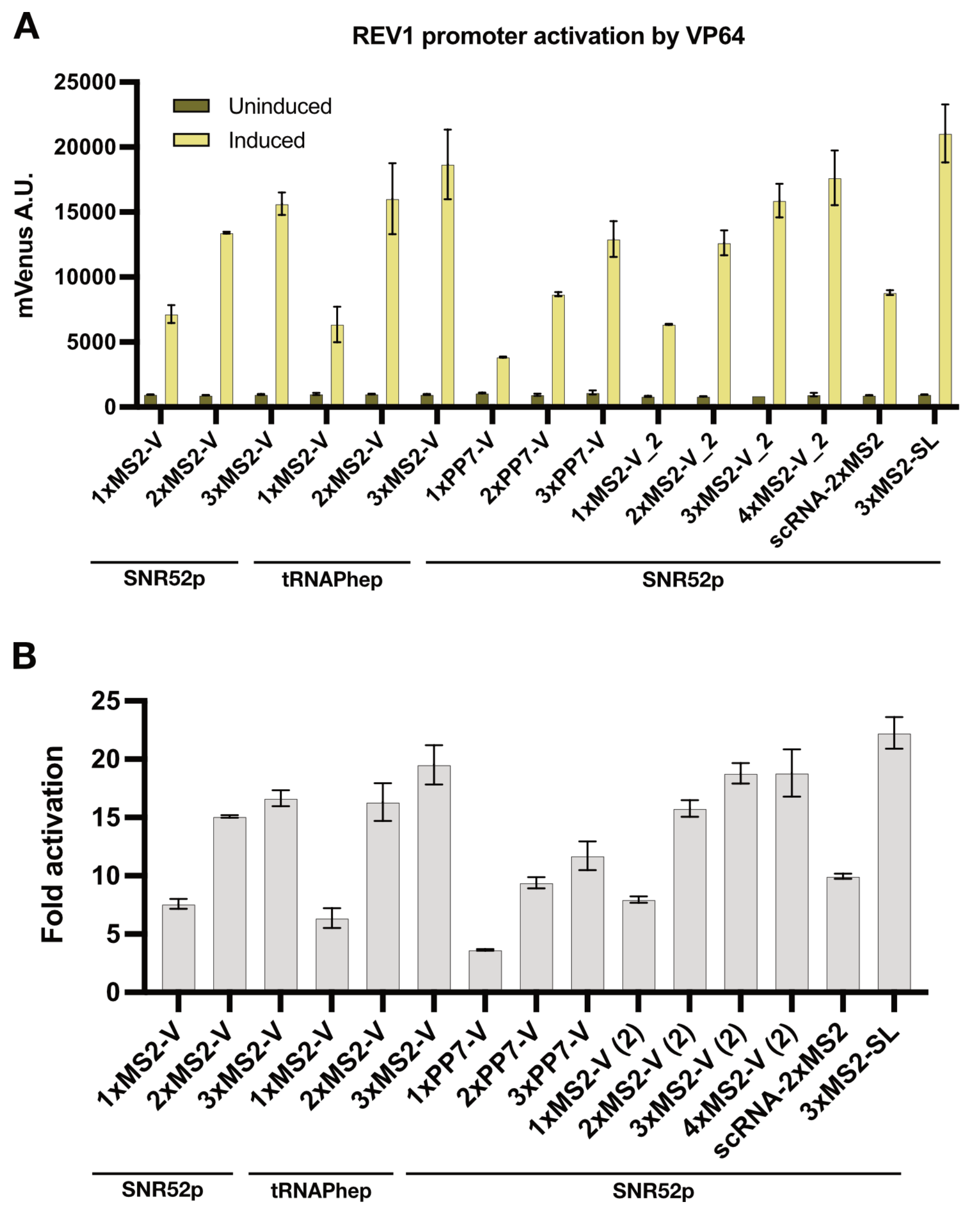

Supplementary Figure 3. Comparison of sgRNAOs with previously reported designs.
(A) REV1 promoter activation by VP64 in strains expressing sgRNAOs, scRNA-2xMS2 or 3xMS2-SL upon induction of the CRISPR machinery verified by the expression of the mVenus fluorescence protein reporter in yeast. 3-helix sgRNAO constructs carrying up to three MS2 or PP7 hairpins are shown, transcribed using either the SNR52 or tRNA Phe expression strategies. The scRNA-2xMS2 is obtained by Zalatan *et al.* and the 3xMS2-SL is obtained by Shechner *et al.*[1, 2]. Data obtained using flow cytometry. Mean values and ±SD from biological triplicates are shown. **(B)** Relative fold activation ±SEM of the REV1 promoter by VP64 after induction, calculated using the data shown on panel (A).

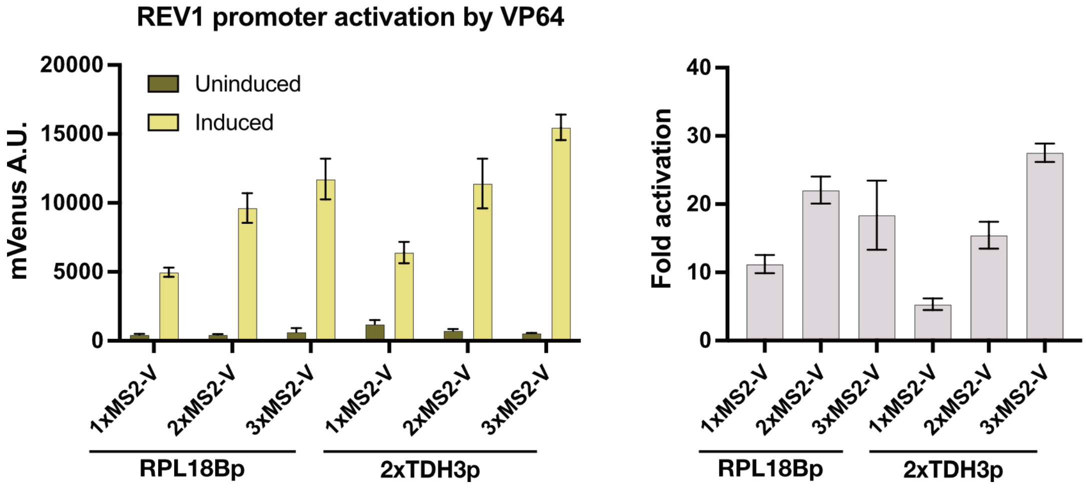

Supplementary Figure 4. REV1 promoter activation when varying MCP-VP64 expression.

REV1 promoter activation by VP64 in strains expressing sgRNAOs upon induction of the CRISPR machinery verified by the expression of the mVenus fluorescence protein reporter in yeast. 3-helix sgRNAO constructs carrying up to three MS2 are shown, transcribed using the SNR52 expression strategy. The MCP-VP64 protein motif is expressed by either a weak RPL18 promoter or two expression cassettes driven by the TDH1 promoter. Mean values and ±SD from biological triplicates are shown apart from 1xMS2-V (RPL18Bp) and 3xMS2-V (2xTDH3p) which are shown from duplicates. Relative fold activation ±SEM of the REV1 promoter by VP64 after induction also shown. Data obtained using flow cytometry.

RNA blueprints and sequences

Supplementary Table 1. MS2, PP7 and 3WJ motifs used in this study. The 3WJ-motif image is adapted by Severcan *et al*, 2009[3].

Name	RNA motif
MS2 aptamer	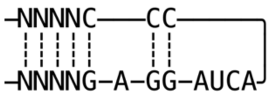
PP7 aptamer	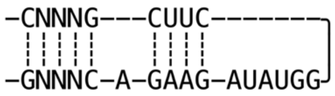
3WJ motif	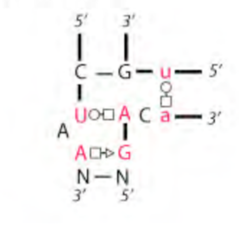

Supplementary Table 2. RNA sequences and 2D blueprints of RNA origami tiles and sgRNAOs.

1xMS2-V
GGAAUAGCUGGAGCGCGGUUAUAUCCAACGAAAGUUGGCGGAUUCCCUUCGGGGAAUCGUGUGACAUG AGGAUCACCCAUGUUAUGCGGAUUAGGAUGAAGCGAGCACGUUCUAAUCCCGGGCAGGCAUUGAAGCC UCCACGAUGCCUGUCAUAUAGCCGCGUUCCAGCUGUUCCCCGUGUAUGCGCAUGCCGCCUGAAGGAG GCACGGGCGGUAUGGGGCAGUUCUAUGAAGCUCGCACGUAGGGCUGCCCGGUCCUUUAGAAAUAGAG GACCCGACAAUGGUUCGCCAUUGUGAUCGGAAACGAUCCAUAUACGG
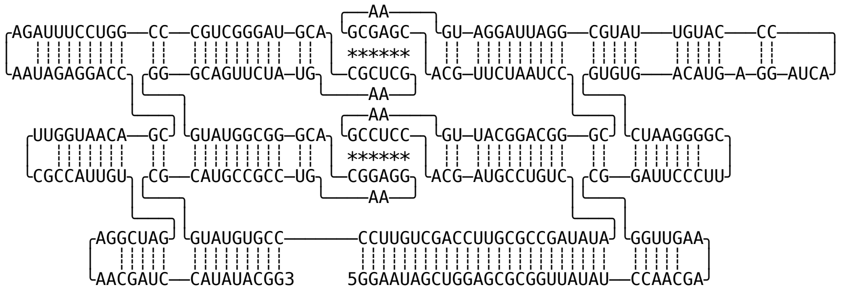

2xMS2-V
GGAAUAGCUGCAUCAGGUGUGCAGCGCUGACAUGAGGAUCACCCAUGUCAGUGCGGUCAACGUUCGCG UUGACGAUACACAUGAGGAUCACCCAUGUGUGUCGCUGCGCCCUGAAGCGAGCACGGGGCGUAGCCG GGUGUUCUUUGAAGCCUCCACGAAGAACAUCCUGCGCACCUGGUGCAGCUGUUCCCCGUGUAUGCGC UGUGAAGCUGAAGGAGGCACGGUUUCACAGGGGCCGAAAGUUGAAGCUCGCACGACUUUCGGUCCGC CUCUGAGAAAUCAGAGGCCGAAGCUGAUUCGUCAGCUUGGCUCGAAAGAGCCCAUAUACGG
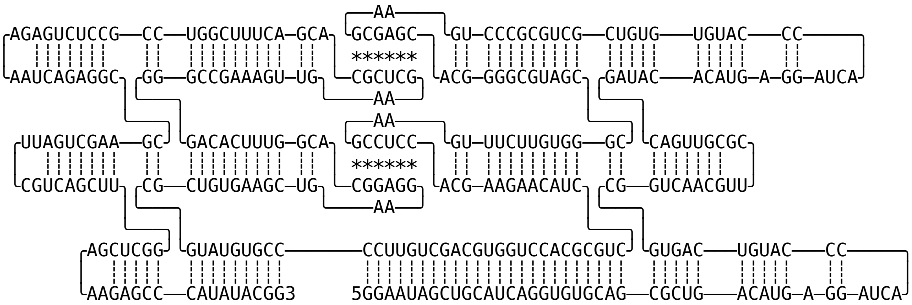

3xMS2-V
GGAAUAGCUGUGAUCAAUUGCACUCCGGUACAUGAGGAUCACCCAUGUAUCGGCGGCUAGGAUUCGUC CUAGCGGUGCACAUGAGGAUCACCCAUGUGCAUCGAGAAUUGCUGAAGCGAGCACGGUAAUUCUCCGG AGUGGUAAUGAAGCCUCCACGUUACUGCUCAGUGCGAUUGAUUACAGCUGUUCCCCGUGUAUGCGCCU UGCGACUGAAGGAGGCACGGUCGUAAGGGGAGCAGUUUGUGAAGCUCGCACGCAAACUGUUCCGGUG UGACAUGAGGAUCACCCAUGUUGCACCCGACUACUCUUCGGAGUAGUGGUGAGAAAUCACCCAUAUAC GG
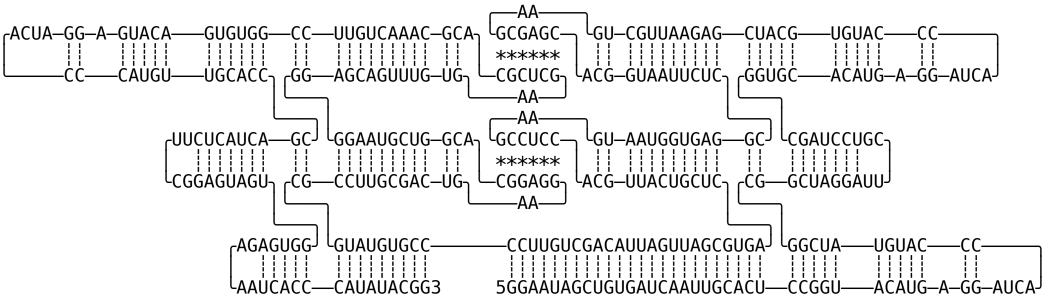

1xPP7

GGAAUAGCUGCGUGCGGGUUUAGCCUUCCGAAAGGAAGCGGGAACUCUUCGGAGUUCCGGUCCGGCA
CAGAAGAUAUGGCUUCGUGCCGGAUCGCGUAUGGCUGAAGCGAGCACGGCCAUGCGCCGGAAGAGUC
GUGAAGCCUCCACGCGGCUUUUCGCUAGACUCGCACGUAGCUGUUCCCCGUGUAUGCGCAUUCCUGU
UGAAGGAGGCACGGCAGGAGUGGGUGUUUUAGAUGAAGCUCGCACGUUUAGAACACCGGCAAAAUCGA
AAGGUUUUGCCCGACUAAUGUUCGCAUUAGUGGCUCGAAAGAGCCCAUAUACGG

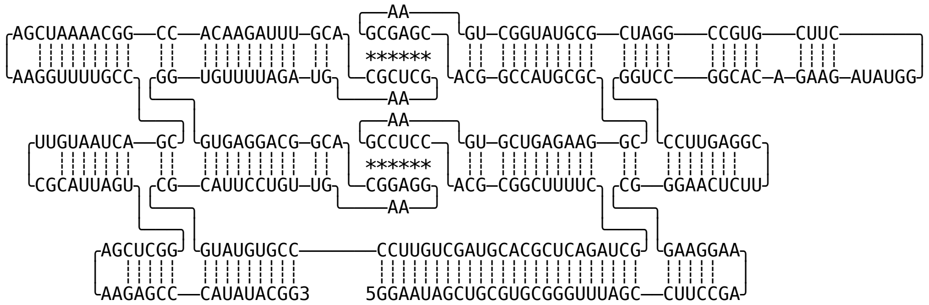

2xPP7

GGAAUAGCUGGGAUGGGGAGCAUGCUAUGGCACAGAAGAUAUGGCUUCGUGCCAUGGCGGUGGGAGU
UCGCUCCCACGAUUCGGUGCAGAAGAUAUGGCUUCGCAUCGGGUCGAAUUAUAGUGAAGCGAGCACGC
UAUGGUUCCGGUUCUGCGCUGAAGCCUCCACGGUGCAGAACCAUGCUUCUUAUUUCAGCUGUUCCCC
GUGUAUGCGCGCAAUUUGUGAAGGAGGCACGCAAAUUGUGGGUAACCGAGCUGAAGCUCGCACGGCUC
GGUUGCCGGUACGUUCGAAAGAGCGUACCCGAAUCGGCUUCGGCCGAUUGUACGGAAACGUACCAUAU
ACGG

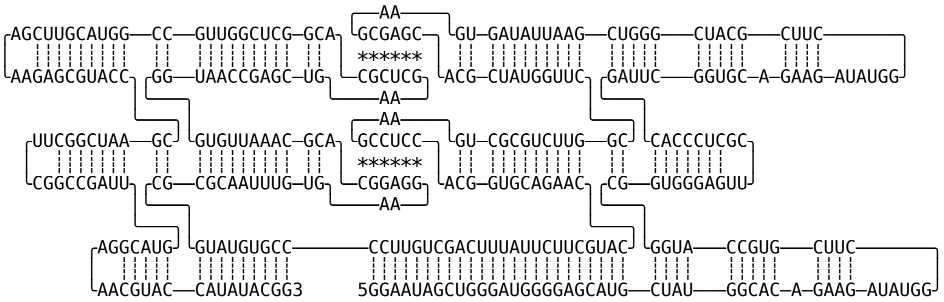

3xPP7

GGAAUAGCUACUGAUCCCUGUUGGCGUGGGCACAGAAGAUAUGGCUUCGUGCCUACGCGGAGAACCUU
CGGGUUCUCGGGUUGAUGCAGAAGAUAUGGCUUCGCAUCAGCCCGUUGCCAUGUGAAGCGAGCACGC
AUGGCAGCCGGAUCCCUUUUGAAGCCUCCACGGAAGGGGUCCCAGCAGGGGUUAGUAGCUGUUCCCC
GUGUAUGCGCAUCUUAGGUGAAGGAGGCACGCCUGAGAUGGGACACUAGCAUGAAGCUCGCACGUGCU
GGUGUCCGGUUGCGAGCCAGAAGAUAUGGCUUCGGCUCGUAACCCGACCCUCGUUCGCGAGGGUGAU
UCGAAAGAAUCCAUAUACGG

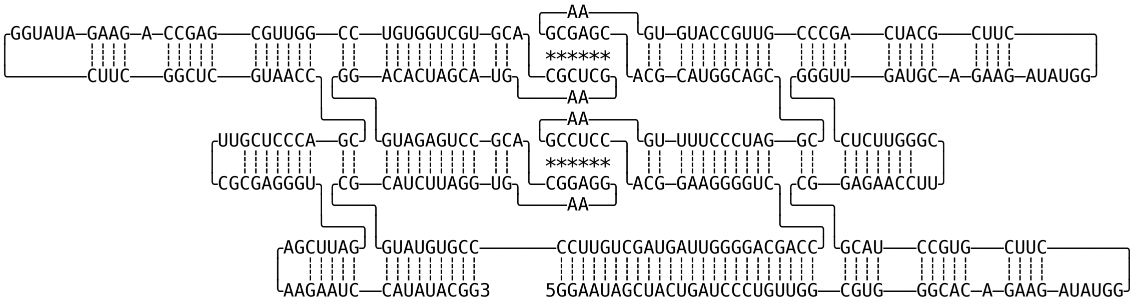

1xMS2-V_2/1xMS2-V-B
GGAAUAGCUGGUGCCUGACGUGAGCGCGUGAAAACGUGCGGACCAUCUUCGGAUGGUCGAUUGACAUGAGGAUCACCCAUGUCGGUCGCGUGAAGCUGAAGCGAGCACGGCUUCACGCCGGCUAACCCGUGAAGCCUCCACGCGGGUUAGCUUCACGUUAGGCACUAGCUGUUCCCCGUGUAUGCGCGUACUGCGUGAAGGAGGCACGCGCAGUACGGGAGCACGCUGUGAAGCUCGCACGCAGCGUGUUCCGGUGAGAAAUCGCCCGACAGAAUUUCGAUUCUGUGUGGCGAAAGUCACCAUAUACGG
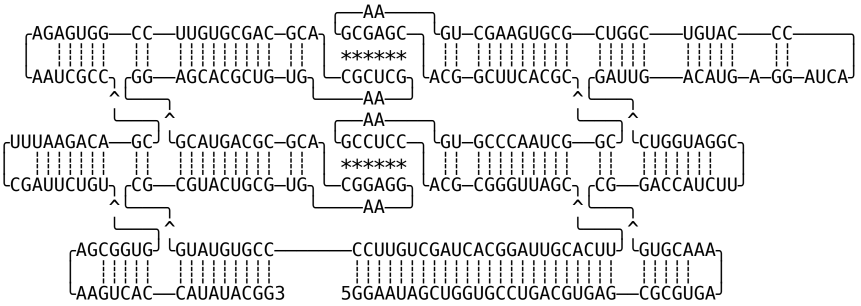

2xMS2-V_2/2-MS2-V-AB
GGAAUAGCUGGUGCCUGACGUGAGCGCGUACAUGAGGAUCACCCAUGUACGUGCGGACCAUCUUCGGAUGGUCGAUUGACAUGAGGAUCACCCAUGUCGGUCGCGUGAAGCUGAAGCGAGCACGGCUUCACGCCGGCUAACCCGUGAAGCCUCCACGCGGGUUAGCUUCACGUUAGGCACUAGCUGUUCCCCGUGUAUGCGCGUACUGCGUGAAGGAGGCACGCGCAGUACGGGAGCACGCUGUGAAGCUCGCACGCAGCGUGUUCCGGUGAGAAAUCGCCCGACAGAAUUUCGAUUCUGUGUGGCGAAAGUCACCAUAUACGG
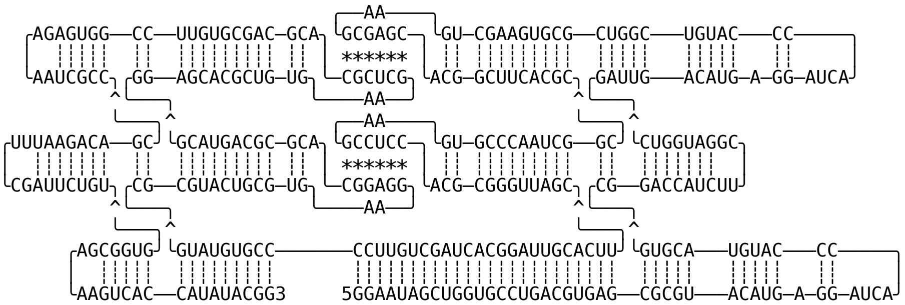

3xMS2-V_2
GGAAUAGCUGGUGCCUGACGUGAGCGCGUACAUGAGGAUCACCCAUGUACGUGCGGACCAUCUUCGGAUGGUCGAUUGACAUGAGGAUCACCCAUGUCGGUCGCGUGAAGCUGAAGCGAGCACGGCUUCACGCCGGCUAACCCGUGAAGCCUCCACGCGGGUUAGCUUCACGUUAGGCACUAGCUGUUCCCCGUGUAUGCGCGUACUGCGUGAAGGAGGCACGCGCAGUACGGGAGCACGCUGUGAAGCUCGCACGCAGCGUGUUCCGGUGAACAUGAGGAUCACCCAUGUUCGCCCGACAGAAUUUCGAUUCUGUGUGGCGAAAGUCACCAUAUACGG
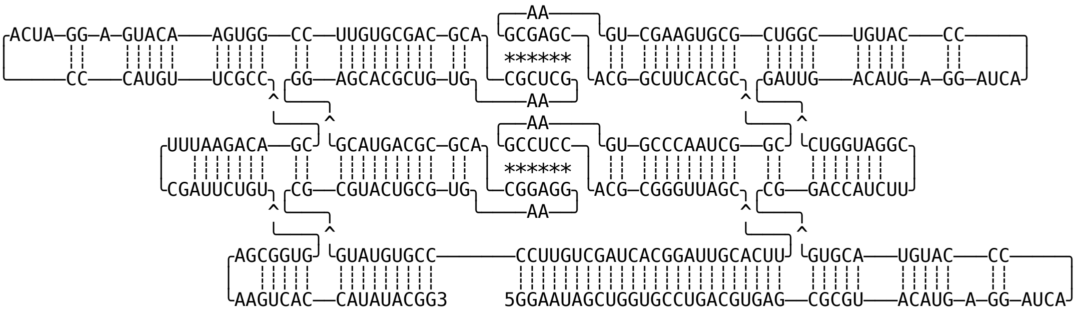

4xMS2-V_2

GGAAUAGCUGGUGCCUGACGUGAGCGCGUACAUGAGGAUCACCCAUGUACGUGCGGACCAUCUUCGGA
UGGUCGAUUGACAUGAGGAUCACCCAUGUCGGUCGCGUGAAGCUGAAGCGAGCACGGCUUCACGCCG
GCUAACCCGUGAAGCCUCCACGCGGGUUAGCUUCACGUUAGGCACUAGCUGUUCCCCGUGUAUGCGC
GUACUGCGUGAAGGAGGCACGCGCAGUACGGGAGCACGCUGUGAAGCUCGCACGCAGCGUGUUCCGG
UGAACAUGAGGAUCACCCAUGUUCGCCCGACAGAAUUUCGAUUCUGUGUGGCACAUGAGGAUCACCCA
UGUGUCACCAUAUACGG

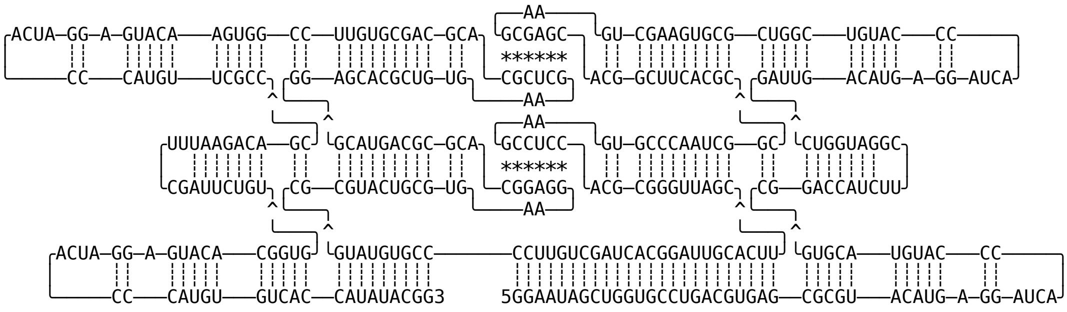

1xMS2-V-A

GGAAUAGCUGGUGCCUGACGUGAGCGCGUACAUGAGGAUCACCCAUGUACGUGCGGACCAUCUUCGGA
UGGUCGAUUGGAAACGGUCGCGUGAAGCUGAAGCGAGCACGGCUUCACGCCGGCUAACCCGUGAAGC
CUCCACGCGGGUUAGCUUCACGUUAGGCACUAGCUGUUCCCCGUGUAUGCGCGUACUGCGUGAAGGA
GGCACGCGCAGUACGGGAGCACGCUGUGAAGCUCGCACGCAGCGUGUUCCGGUGAGAAAUCGCCCGA
CAGAAUUUCGAUUCUGUGUGGCGAAAGUCACCAUAUACGG

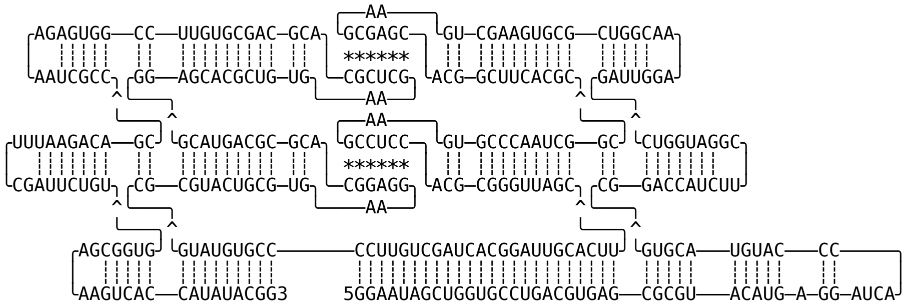

1xMS2-V-C

GGAAUAGCUGGUGCCUGACGUGAGCGCGUGAAAACGUGCGGACCAUCUUCGGAUGGUCGAUUGGAAAC
GGUCGCGUGAAGCUGAAGCGAGCACGGCUUCACGCCGGCUAACCCGUGAAGCCUCCACGCGGGUUAG
CUUCACGUUAGGCACUAGCUGUUCCCCGUGUAUGCGCGUACUGCGUGAAGGAGGCACGCGCAGUACG
GGAGCACGCUGUGAAGCUCGCACGCAGCGUGUUCCGGUGAACAUGAGGAUCACCCAUGUUCGCCCGA
CAGAAUUUCGAUUCUGUGUGGCGAAAGUCACCAUAUACGG

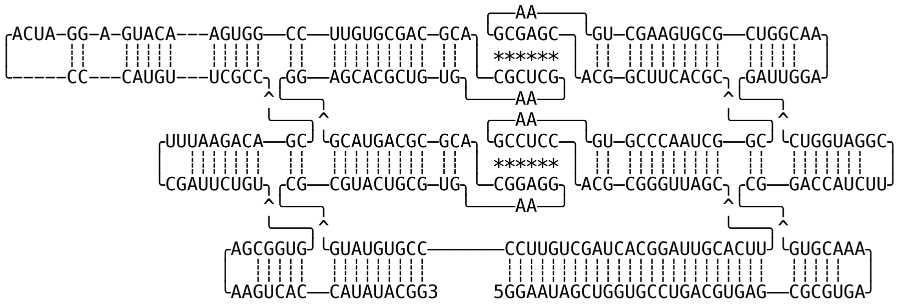

1xMS2-V-D

GGAAUAGCUGGUGCCUGACGUGAGCGCGUGAAAACGUGCGGACCAUCUUCGGAUGGUCGAUUGGAAAC
GGUCGCGUGAAGCUGAAGCGAGCACGGCUUCACGCCGGCUAACCCGUGAAGCCUCCACGCGGGUUAG
CUUCACGUUAGGCACUAGCUGUUCCCCGUGUAUGCGCGUACUGCGUGAAGGAGGCACGCGCAGUACG
GGAGCACGCUGUGAAGCUCGCACGCAGCGUGUUCCGGUGAGAAAUCGCCCGACAGAAUUUCGAUUCUG
UGUGGCACAUGAGGAUCACCCAUGUGUCACCAUAUACGG

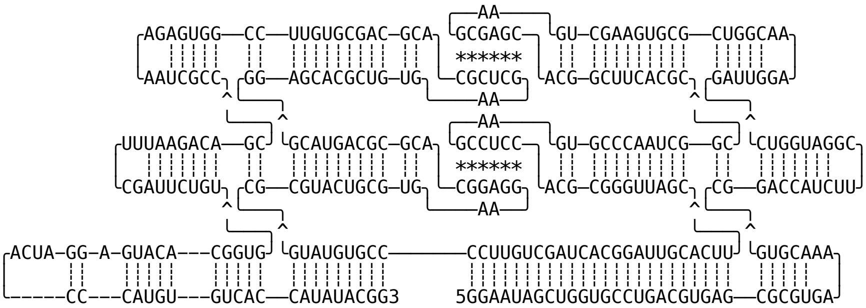

2xMS2-V-BC

GGAAUAGCUGGUGCCUGACGUGAGCGCGUGAAAACGUGCGGACCAUCUUCGGAUGGUCGAUUGACAU
GAGGAUCACCCAUGUCGGUCGCGUGAAGCUGAAGCGAGCACGGCUUCACGCCGGCUAACCCGUGAAGC
CUCCACGCGGGUUAGCUUCACGUUAGGCACUAGCUGUUCCCCGUGUAUGCGCGUACUGCGUGAAGGA
GGCACGCGCAGUACGGGAGCACGCUGUGAAGCUCGCACGCAGCGUGUUCCGGUGAACAUGAGGAUCA
CCCAUGUUCGCCCGACAGAAUUUCGAUUCUGUGUGGCGAAAGUCACCAUAUACGG

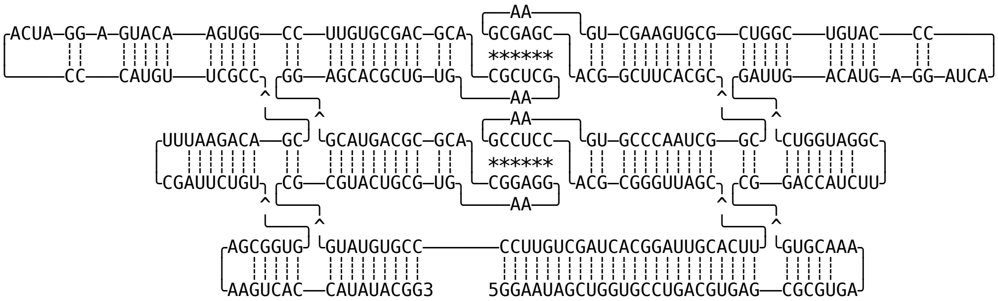

2xMS2-V-CD

GGAAUAGCUGGUGCCUGACGUGAGCGCGUGAAAACGUGCGGACCAUCUUCGGAUGGUCGAUUGGAAAC
GGUCGCGUGAAGCUGAAGCGAGCACGGCUUCACGCCGGCUAACCCGUGAAGCCUCCACGCGGGUUAG
CUUCACGUUAGGCACUAGCUGUUCCCCGUGUAUGCGCGUACUGCGUGAAGGAGGCACGCGCAGUACG
GGAGCACGCUGUGAAGCUCGCACGCAGCGUGUUCCGGUGAACAUGAGGAUCACCCAUGUUCGCCCGA
CAGAAUUUCGAUUCUGUGUGGCACAUGAGGAUCACCCAUGUGUCACCAUAUACGG

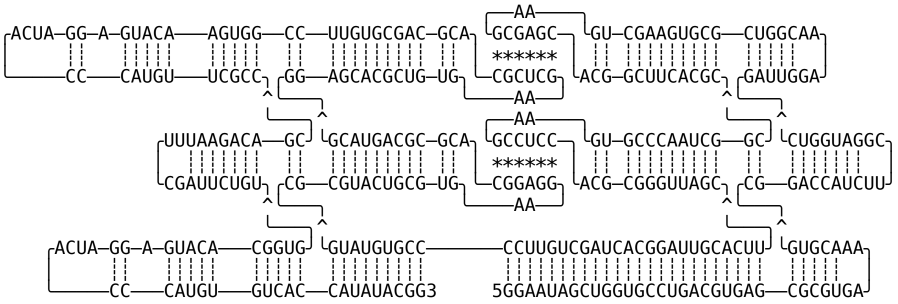

2xMS2-1xPP7-V

GGAAUAGCUACGCGCUAAGUCUAGCCACCGAAAGGUGGCGGUGAGUUCGCUUACGCUUGGAAACAAGC
GAACUGCCAUGAAGCGAGCACGUGGCAGUUCCGGGGUAUUGCUGAAGCCUCCACGGCAAUACUCCUGG
ACUUGGUGCGUAGCUGUUCCCCGUGUAUGCGCACUCCCCGUGAAGGAGGCACGCGGGGAGUGGGGAC
AAGUGUUGAAGCUCGCACGACGCUUGUCCCGUCGUACAUGAGGAUCACCCAUGUGCGACCGAGCUUGG
CACAGAAGAUAUGGCUUCGUGCCAAGUUGCCUGACAUGAGGAUCACCCAUGUCGGGCCAUAUACGG

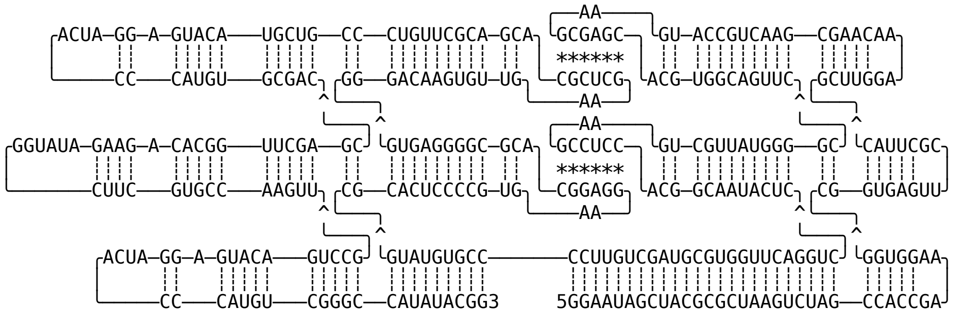

2xMS2-0xPP7-V

GGAAUAGCUACGCGCUAAGUCUAGCCACCGAAAGGUGGCGGUGAGUUCGCUUACGCUUGGAAACAAGC
GAACUGCCAUGAAGCGAGCACGUGGCAGUUCCGGGGUAUUGCUGAAGCCUCCACGGCAAUACUCCUGG
ACUUGGUGCGUAGCUGUUCCCCGUGUAUGCGCACUCCCCGUGAAGGAGGCACGCGGGGAGUGGGGAC
AAGUGUUGAAGCUCGCACGACGCUUGUCCCGUCGUACAUGAGGAUCACCCAUGUGCGACCGAGCUUUU
CGAAGUUGCCUGACAUGAGGAUCACCCAUGUCGGGCCAUAUACGG

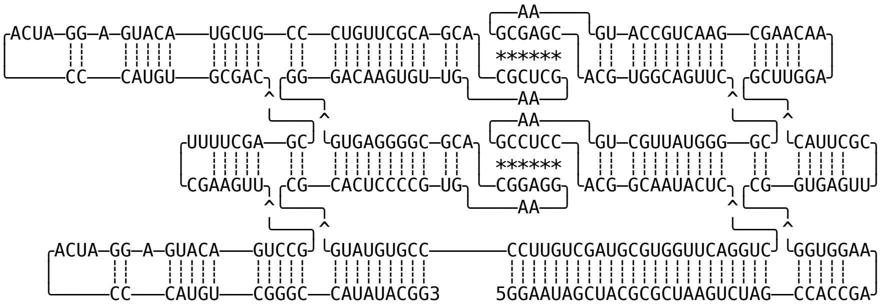

2xPP7-1xMS2-V

GGAAUAGCUAGCGCCUGCACCCGCCUAUGGAAACAUAGCGGGGUGUUCGUACCCGCAGCGAAAGCUGC
GCGAUAAGUUGAAGCGAGCACGACUUAUCGCCGGUAGGAACGUGAAGCCUCCACGCGUUCCUGCGCGG
GUGCGGGUGCUAGCUGUUCCCCGUGUAUGCGCCAAUCGUGUGAAGGAGGCACGCACGAUUGGGGAUG
GGUCGAUGAAGCUCGCACGUCGACCUAUCCGAUAUGGCACAGAAGAUAUGGCUUCGUGCCAUGUCCGA
UCUCACAUGAGGAUCACCCAUGUGGGAUGUCAUGGGGCAGAAGAUAUGGCUUCGCUCCAUGACCAUAU
ACGG

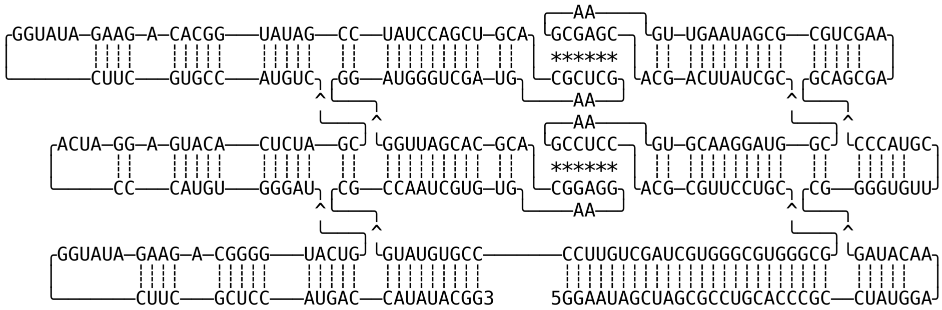

2xPP7-1xMS2-V-NC

GGAAUAGCUAGCGCCUGCACCCGCCUAUGGAAACAUAGCGGGGUGACAUGAGGAUCACCCAUGUUACC
CGCAGCGAAAGCUGCGCGAUAAGUUGAAGCGAGCACGACUUAUCGCCGGUAGGAACGUGAAGCCUCCA
CGCGUUCCUGCGCGGGUGCGGGUGCUAGCUGUUCCCCGUGUAUGCGCCAAUCGUGUGAAGGAGGCAC
GCACGAUUGGGGAUGGGUCGAUGAAGCUCGCACGUCGACCUAUCCGAUAUGGCACAGAAGAUAUGGCU
UCGUGCCAUGUCCGAUCUCUUCGGGGAUGUCAUGGGGCAGAAGAUAUGGCUUCGCUCCAUGACCAUA
UACGG

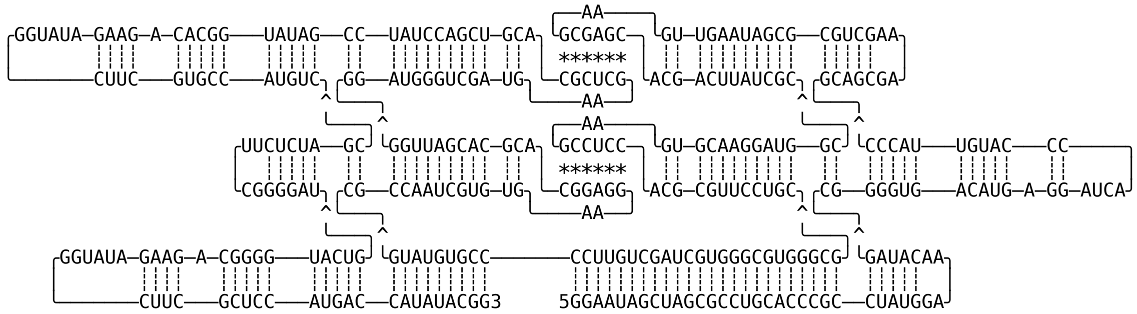

2xPP7-0xMS2-V

GGAAUAGCUAGCGCCUGCACCCGCCUAUGGAAACAUAGCGGGGUGUUCGUACCCGCAGCGAAAGCUGC
GCGAUAAGUUGAAGCGAGCACGACUUAUCGCCGGUAGGAACGUGAAGCCUCCACGCGUUCCUGCGCGG
GUGCGGGUGCUAGCUGUUCCCCGUGUAUGCGCCAAUCGUGUGAAGGAGGCACGCACGAUUGGGGAUG
GGUCGAUGAAGCUCGCACGUCGACCUAUCCGAUAUGGCACAGAAGAUAUGGCUUCGUGCCAUGUCCGA
UCUCUUCGGGGAUGUCAUGGGGCAGAAGAUAUGGCUUCGCUCCAUGACCAUAUACGG

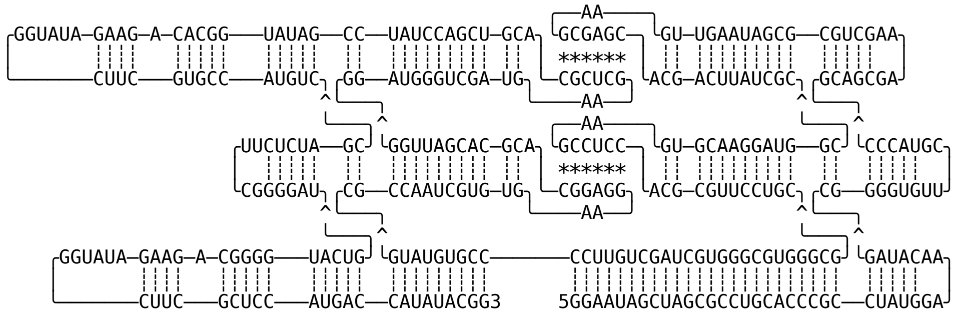

3xMS2-H

GUUUAAGAGCUAUGCUGCGAAUACGAGGCAUGGGAUCAUUCUGUGUGGCGAAAGUCACCAUAUACGGG
GAAUAGCUGGUGCCUGACGUGAGCGCGUACAUGAGGAUCACCCAUGUACGUGCGGACCAUCUUCGGAU
GGUCGAUUGACAUGAGGAUCACCCAUGUCGGUCGCGUGAAGCUGAAGCGAGCACGGCUUCACGCCGG
CUAACCCGUGAAGCCUCCACGCGGGUUAGCUUCACGUUAGGCACUAGCUGUUCCCCGUGUAUGCGCG
UACUGCGUGAAGGAGGCACGCGCAGUACGGGAGCACGCUGUGAAGCUCGCACGCAGCGUGUUCCGGU
GAACAUGAGGAUCACCCAUGUUCGCCCGACAGAAUGGUUCCAUGUCUCGUAUUCGCAGCAUAGCAAGU
UUAAAUAAGGCUAGUCCGUUAUCAACUUGAAAAAGUGGCACCGAGUCGGUGC

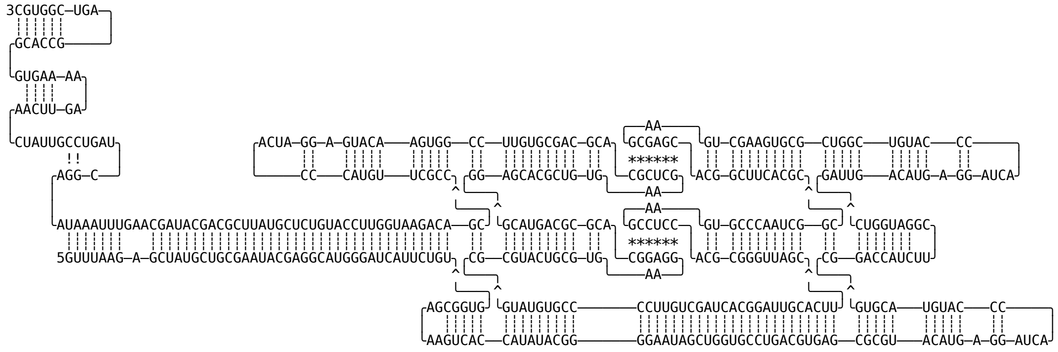

sgRNA-1xMS2-3WJ
GUUUAAGAGCUAUGCUGCGAAUACGAGCACUGAAGGGAUAGGCGGCUUAGGGGCUAUCGAAAGAUAGG<br>GGACAAACUUCGGUUUGUCGUGUGACAUGAGGAUCACCCAUGUCACGCCGUCCUCCCUGAAGCGAGCA<br>CGGGGAGGACGCCGAACUCCCGUGAAGCCUCCACGCGGGAGUUCCCUCUAAGUCGUCUGUCUCUUCU<br>AACGCGCUAGCCCCGGUCACUUGAAGGAGGCACGAGUGACCGGCCCAGUCUGGCUGAAGCUCGCACG<br>GCUAGACUGGGGCUCUUAUGGAAACAUAAGAGCGGGUUUUGUUUCGACAAAACGCUGCGAAAGCAGCC<br>UAGCGCGGACAGUGCUCGUAUUCGCAGCAUAGCAAGUUUAAAUAAGGCUAGUCCGUUAUCAACUUGAA<br>AAAGUGGCACCGAGUCGGUGC
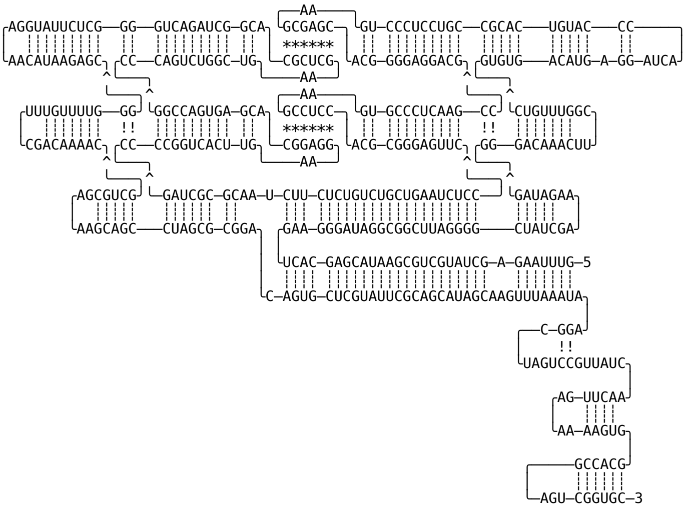

sgRNA-4xMS2-3WJ
GUUUAAGAGCUAUGCUGCGAAUACGAGCACUGAAAGAAUAUCCAUGUGUAGAGCCCUUGACAUGAGGA<br>UCACCCAUGUUAAGGCCGUCGCGAUUCGUCGCGACCGAUUUGAUGAGGAUCACCCAUCAGAUCGCUAU<br>UGUUUUGAAGCGAGCACGAAACAAUAGGGGCCUGAACUUGAAGCCUCCACGAGUUCAGGCGCUCUGCA<br>CAUGGAUGUUCUUUCUAACGUUCGGCGGCUGGGGAGUUGAAGGAGGCACGACUCCCCAGGGUUUCUU<br>CCGUGAAGCUCGCACGCGGAAGGAACCGGAGCUAGAAGAGGAUCACCCUUCUAGUUCCCCGGCUACGU<br>UCGCGUAGCCCUGGUGUAUGAGGAUCACCCAUACACUAGGCCGAACGGACAGUGCUCGUAUUCGCAGC<br>AUAGCAAGUUUAAAUAAGGCUAGUCCGUUAUCAACUUGAAAAAGUGGCACCGAGUCGGUGC
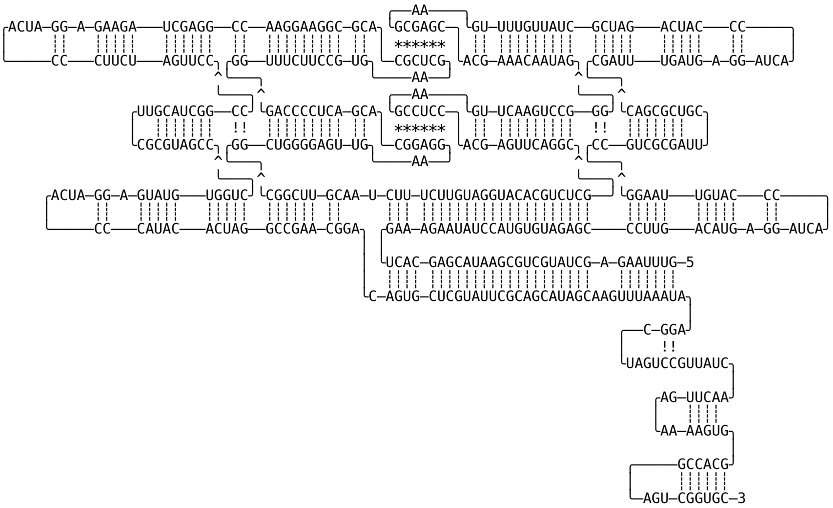

sgRNA-4xMS2-H

GUUUAAGAGCUAUGCUGCGAAUACGAGUUGUUACCGCAUUUCCCCAUGAGUAUGAGGAUCACCCAUAC
UUAUGCGUUCCAGUUCGGAAGCUGGAGGGGCAACGAGCCUUGGACAUGAGGAUCACCCAUGUUCAAGC
CGGCCUCCUUCGGGAGGCCCCGAUUGAUGAGGAUCACCCAUCAGUCGGGCACCUUACUGAAGCGAGCA
CGGUAAGGUGCGGGAGGAGCUCUGAAGCCUCCACGGAGCUCCUCGCUCGUUGCUCCUUCAGCUUUCG
AACUGGAGCGGGCGACGCCGUUGAAGGAGGCACGACGGCGUCGGGGCGAAUGAAUGAAGCUCGCACG
UUUAUUCGCCCGGUUGCAGAAGAGGAUCACCCUUCUGCGACCCCGGGAAAUGUGGUGAUAGCUCGUAU
UCGCAGCAUAGCAAGUUUAAAUAAGGCUAGUCCGUUAUCAACUUGAAAAAGUGGCACCGAGUCGGUGC

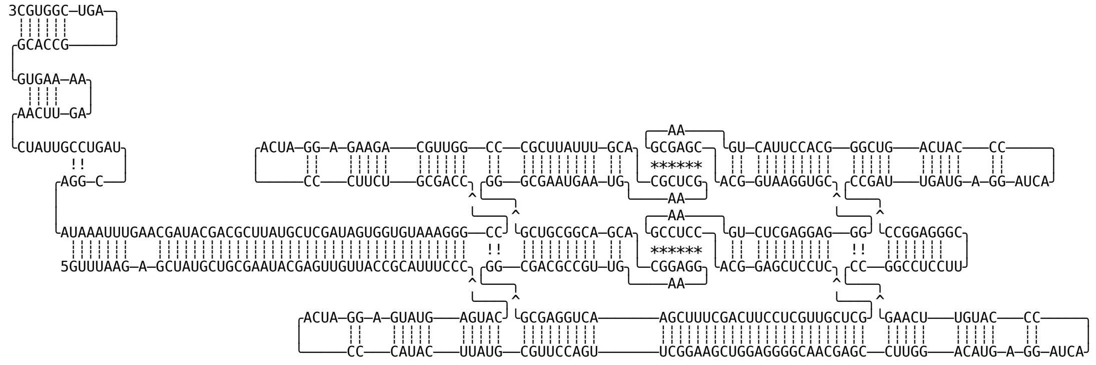

4xMS2-V-5Hv.1

GGAACUCAUUUCGCCCACCCUCAUACAUGAGGAUCACCCAUGUAUGGGCGGAGGUUGUUCGCAACCUC
GGCAGUCCUGUGGUGCAGUGGGAAACCGCUGCACCCGGUAGGCAUUCGUGCCUACUCUCCACAUGAG
GAUCACCCAUGUGGGGAGACUACCGCACAACCCGAAAGUGCGGUGGUCCGGCUGCCAGGGAAAUAGAC
GAUCUCUGGCAGCAUAGGACUGCCAACGACAAUCACUCAGUCGUUCGUUACUUUGACAAACACACGAU
GUCGAAGUAAGGUGGGUGGGAUGAGUUCCCCGUGUAUGGCGUAGGCUAUAGGCGGCAACGUGUGAGC
CGCCUGUAGGAGUCGGUGUGGGCACCGAAGAGUGAACGGUGCCGCAUAGUGUCUGCAACGUCUAAGU
AGACGCUAUCGUGUCCAAUGGUAAUUCGGGAACCAUUGGGCACGCGGCGACAUGAGGAUCACCCAUGU
CGUCGGCAGGUUGUUUCGACAACCUCACGCCGACUCGUCGCUUCAUGAAAAUGAAGUGGCCCAACAGU
AUUCGUACUGUUCUUGAACAUGAGGAUCACCCAUGUUCGAGUGCGUCAUACACGG

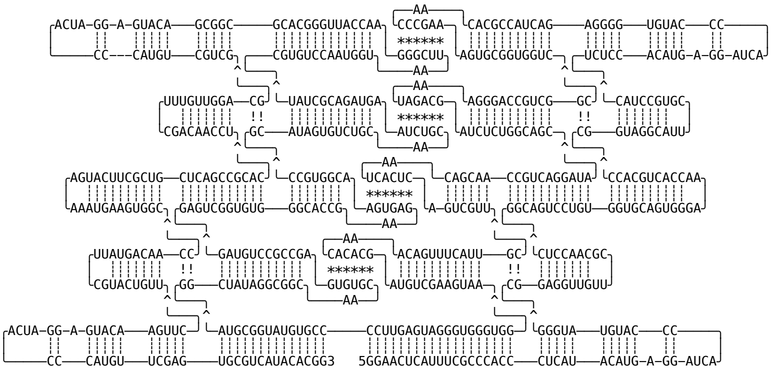

4xMS2-V-5Hv.2
GGAACUCAUUCCUUACAUUAUUUGACAUGAGGAUCACCCAUGUCGAAUCGUUCCCCAUUCGUGGGGAA AGCGGUGGCUCAGAUUCCUCUGAAAAGAGGGAUCUCCAACGUUAUUCGUAACGUUCGUGCACAUGAGG AUCACCCAUGUGUACGUAGUGGCGCGAAAUUCGUCAUCGCGUCACUAGGCAGAAUGCGCAAAUCUCGC AUGCGUAUUCUGGAGCCAUCGCUCUGGCCAAUCACUGAGGCCAGCGGGAGGUGCAAUAAGAGGUGAAU UGCGCCUCCAAUGUGAGGGAUGAGUUCCCCGUGUAUGGCUGCCCCGCAGCUUUGUAACACCUCAACAA AGUUGCGCCUGGCCUGUUCCUGAGCAACAGUGAAGCUCAGGCGCCCUCUGGCUUAAGCGAGAAAAGCC GGAGGGGCAGGGUAAAGCAAAGACGAAAUGCUUUGCCCUGCAUUUUACAUGAGGAUCACCCAUGUAGG AUCGUAACCAGUUCGCUGGUUAAACAGGUCAGGGGAUGGCGCUGAAAAGCGCUAUCCGGACUCAGAUU CGUCUGAGUGAUCAACAUGAGGAUCACCCAUGUUGGUCGUAGUCAUACACGG
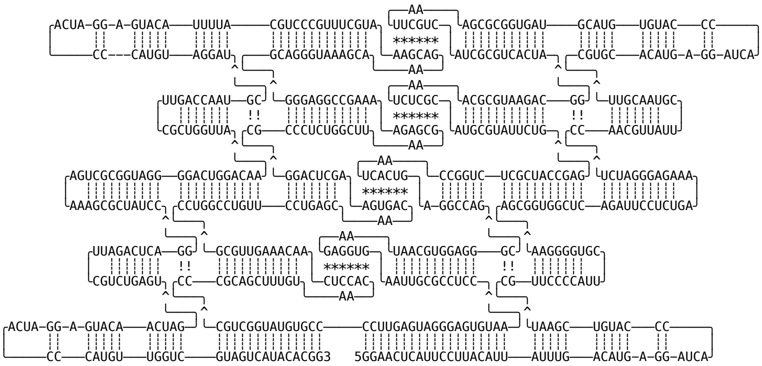

Plasmids

Supplementary Table 3. Yeast plasmids created for sgRNA-O mediated REV1 upregulation. Promoters are shown in red.

Name	Yeast marker	Gene 1	Gene 2	Gene 3	Gene 4
pGPY572	*LEU2*	dCas9-NLS (LXp)	LacI (PGK1p)	_	_
pGPY576	*URA3*	1xMS2-V (SNR52p)	mVenus (REV1p)	NLS-MCP-VP64 (TDH3p)	_
pGPY577	*URA3*	3xMS2-V (SNR52p)	mVenus (REV1p)	NLS-MCP-VP64 (TDH3p)	_
pGPY580	*URA3*	1xMS2-V ($tRNA^{Phe}$p)	mVenus (REV1p)	NLS-MCP-VP64 (TDH3p)	_
pGPY581	*URA3*	3xMS2-V ($tRNA^{Phe}$p)	mVenus (REV1p)	NLS-MCP-VP64 (TDH3p)	_
pGPY583	*URA3*	2xMS2-V (SNR52p)	mVenus (REV1p)	NLS-MCP-VP64 (TDH3p)	_
pGPY585	*URA3*	2xMS2-V ($tRNA^{Phe}$p)	mVenus (REV1p)	NLS-MCP-VP64 (TDH3p)	_
pGPY598	*URA3*	1xPP7-V (SNR52p)	mVenus (REV1p)	NLS-PCP-VP64 (TDH3p)	_
pGPY600	*URA3*	2xPP7-V (SNR52p)	mVenus (REV1p)	NLS-PCP-VP64 (TDH3p)	_
pGPY602	*URA3*	3xPP7-V (SNR52p)	mVenus (REV1p)	NLS-PCP-VP64 (TDH3p)	_
pGPY604	*URA3*	3xMS2-SL[2] (SNR52p)	mVenus (REV1p)	NLS-MCP-VP64 (TDH3p)	_
pGPY656	*URA3*	4xMS2-3WJ (SNR52p)	mVenus (REV1p)	NLS-MCP-VP64 (TDH3p)	_
pGPY660	*URA3*	1xMS2-3WJ (SNR52p)	mVenus (REV1p)	NLS-MCP-VP64 (TDH3p)	_
pGPY664	*URA3*	4xMS2-H (SNR52p)	mVenus (REV1p)	NLS-MCP-VP64 (TDH3p)	_
pGPY704	*URA3*	4xMS2-V-5Hv.1 ($tRNA^{Phe}$p)	mVenus (REV1p)	NLS-MCP-VP64 (TDH3p)	_
pGPY708	*URA3*	4xMS2-V-5Hv.2 ($tRNA^{Phe}$p)	mVenus (REV1p)	NLS-MCP-VP64 (TDH3p)	_
pGPY776	*URA3*	1xMS2-V (SNR52p)	mVenus (REV1p)	NLS-MCP-VP64 (RPL18Bp)	
pGPY777	*URA3*	2xMS2-V (SNR52p)	mVenus (REV1p)	NLS-MCP-VP64 (RPL18Bp)	
pGPY778	*URA3*	3xMS2-V (SNR52p)	mVenus (REV1p)	NLS-MCP-VP64 (RPL18Bp)	
pGPY780	*URA3*	1xMS2-V (SNR52p)	mVenus (REV1p)	NLS-MCP-VP64 (TDH3p)	NLS-MCP-VP64 (TDH3p)
pGPY781	*URA3*	2xMS2-V (SNR52p)	mVenus (REV1p)	NLS-MCP-VP64 (TDH3p)	NLS-MCP-VP64 (TDH3p)
pGPY782	*URA3*	3xMS2-V (SNR52p)	mVenus (REV1p)	NLS-MCP-VP64 (TDH3p)	NLS-MCP-VP64 (TDH3p)
pGPY784	*URA3*	1xMS2-V-A (SNR52p)	mVenus (REV1p)	NLS-MCP-VP64 (TDH3p)	_

pGPY785	*URA3*	1xMS2-V-B (SNR52p)	mVenus (REV1p)	NLS-MCP-VP64 (TDH3p)	_
pGPY786	*URA3*	1xMS2-V-C (SNR52p)	mVenus (REV1p)	NLS-MCP-VP64 (TDH3p)	_
pGPY787	*URA3*	1xMS2-V-D (SNR52p)	mVenus (REV1p)	NLS-MCP-VP64 (TDH3p)	_
pGPY788	*URA3*	2xMS2-V-CD (SNR52p)	mVenus (REV1p)	NLS-MCP-VP64 (TDH3p)	_
pGPY789	*URA3*	2xMS2-V-BC (SNR52p)	mVenus (REV1p)	NLS-MCP-VP64 (TDH3p)	_
pGPY791	*URA3*	2xMS2-V-AB (SNR52p)	mVenus (REV1p)	NLS-MCP-VP64 (TDH3p)	_
pGPY792	*URA3*	3xMS2-H (SNR52p)	mVenus (REV1p)	NLS-MCP-VP64 (TDH3p)	_
pGPY793	*URA3*	3xMS2-V (SNR52p)	mVenus (REV1p)	NLS-MCP-VP64 (TDH3p)	_
pGPY794	*URA3*	4xMS2-V_2 (SNR52p)	mVenus (REV1p)	NLS-MCP-VP64 (TDH3p)	_
pGPY796	*URA3*	scRNA-2xMS2 (SNR52p)	mVenus (REV1p)	NLS-MCP-VP64 (TDH3p)	_
pGPY797	*URA3*	2xMS2-1xPP7-V (SNR52p)	mVenus (REV1p)	NLS-PCP (TDH3p)	NLS-MCP-VP64 (TDH3p)
pGPY799	*URA3*	2xMS2-0xPP7-V (SNR52p)	mVenus (REV1p)	NLS-PCP (TDH3p)	NLS-MCP-VP64 (TDH3p)
pGPY800	*URA3*	2xPP7-1xMS2-V (SNR52p)	mVenus (REV1p)	NLS-MCP (TDH3p)	NLS-PCP-VP64 (TDH3p)
pGPY801	*URA3*	2xPP7-1xMS2-V-NC (SNR52p)	mVenus (REV1p)	NLS-MCP (TDH3p)	NLS-PCP-VP64 (TDH3p)
pGPY802	*URA3*	2xPP7-0xMS2-V (SNR52p)	mVenus (REV1p)	NLS-MCP (TDH3p)	NLS-PCP-VP64 (TDH3p)

For protein expressing genes, the ADH1 terminator was used.
For sgRNA-Os expressed from the SNR52p, the SUP4 terminator was used.
For sgRNA-Os expressed from the $tRNA^{Phe}$p, the SNR52 terminator was used.

Supplementary Table 4. Yeast plasmids created for violacein pathway regulation. Promoter targets for sgRNA-Os are shown in red.

Name	Yeast marker	Gene 1	Gene 2	Gene 3	Gene 4	Gene 5
pGPY634	*LEU2*	VioA[a]	VioB[b]	VioC[c]	VioD[d]	VioE[e]
pGPY698	*URA3*	dCas9[f]	1xMS2-V-3H[g] (RNR52p)	1xPP7-V-3H[g] (REV1p)	sgRNA[g] (TEF1p)	-
pGPY700	*URA3*	dCas9[f]	-	-	-	-
pGPY702	*URA3*	dCas9[f]	3xPP7-V-3H[g] (REV1p)	-	-	-
pGPY703	*URA3*	dCas9[f]	3xPP7-V-3H[g] (REV1p)	sgRNA[g] (TEF1p)	-	-
pGPY712	*URA3*	dCas9[f]	1xMS2-V-3H[g] (RNR52p)	1xPP7-V-3H[g] (REV1p)	-	-
pGPY713	*URA3*	dCas9[f]	3xMS2-V-3H[g] (RNR52p)	3xPP7-V-3H[g] (REV1p)	-	-

[a]VioA expression is driven by the REV1 promoter and the ADH1 terminator.
[b]VioB expression is driven by the TDH3 promoter and the ADH1 terminator.
[c]VioC expression is driven by the RNR2 promoter and the ADH1 terminator.
[d]VioD expression is driven by the TEF1 promoter and the ADH1 terminator.
[e]VioE expression is driven by the PGK1 promoter and the ADH1 terminator.
[f]dCas9 expression is driven by the TDH3 promoter and the TDH1 terminator.
[g]sgRNA and sgRNA-O expression is driven by the SNR52 promoter and the SUP4 terminator.

References

[1] Zalatan, J. G., Lee, M. E., Almeida, R., Gilbert, L. A., Whitehead, E. H., La Russa, M., Tsai, J. C., Weissman, J. S., Dueber, J. E., Qi, L. S., and Lim, W. A. (2015) Engineering complex synthetic transcriptional programs with CRISPR RNA scaffolds, *Cell 160*, 339-350.

[2] Shechner, D. M., Hacisuleyman, E., Younger, S. T., and Rinn, J. L. (2015) Multiplexable, locus-specific targeting of long RNAs with CRISPR-Display, *Nat Methods 12*, 664-670.

[3] Severcan, I., Geary, C., Verzemnieks, E., Chworos, A., and Jaeger, L. (2009) Square-shaped RNA particles from different RNA folds, *Nano Lett 9*, 1270-1277.
